# ROBO4-Mediated Vascular Integrity Regulates the Directionality of Hematopoietic Stem Cell Trafficking

**DOI:** 10.1016/j.stemcr.2014.12.013

**Published:** 2015-01-29

**Authors:** Stephanie Smith-Berdan, Andrew Nguyen, Matthew A. Hong, E. Camilla Forsberg

**Affiliations:** 1 Institute for the Biology of Stem Cells, Department of Biomolecular Engineering, University of California, Santa Cruz, Santa Cruz, CA 95064, USA

## Abstract

Despite the use of hematopoietic stem cells (HSCs) in clinical therapy for over half a century, the mechanisms that regulate HSC trafficking, engraftment, and life-long persistence after transplantation are unclear. Here, we show that the vascular endothelium regulates HSC trafficking into and out of bone marrow (BM) niches. Surprisingly, we found that instead of acting as barriers to cellular entry, vascular endothelial cells, via the guidance molecule ROBO4, actively promote HSC translocation across vessel walls into the BM space. In contrast, we found that the vasculature inhibits the reverse process, as induced vascular permeability led to a rapid increase in HSCs in the blood stream. Thus, the vascular endothelium reinforces HSC localization to BM niches both by promoting HSC extravasation from blood-to-BM and by forming vascular barriers that prevent BM-to-blood escape. Our results uncouple the mechanisms that regulate the directionality of HSC trafficking and show that the vasculature can be targeted to improve hematopoietic transplantation therapies.

## Highlights

• Endothelial ROBO4 promotes unidirectional HSC trafficking across vessel walls
• Sinusoidal endothelial cells mediate HSC extravasation from blood to bone marrow
• Vascular integrity prevents HSC escape from bone marrow to blood
• Induced vascular permeability rapidly mobilizes HSCs to the blood stream

## Graphical Abstract

**Figure:**
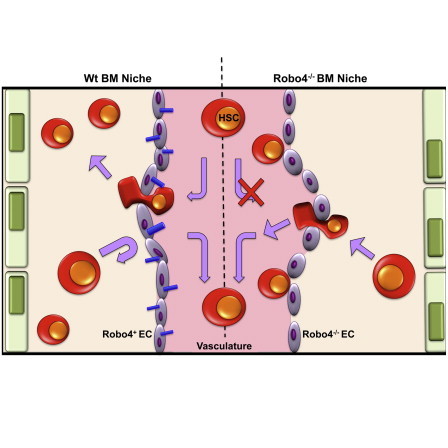

## Teaser

Hematopoietic stem cells (HSCs) depend on a specialized bone marrow environment for optimal life-long function. In this issue, Forsberg and colleagues demonstrate that the vascular endothelium, via the receptor ROBO4, actively promotes HSC translocation from the bloodstream to the bone marrow while also preventing HSC escape from the bone marrow. Their findings uncouple the mechanisms that regulate the directionality of HSC trafficking and point to strategies for manipulating HSC location.

## Introduction

Hematopoietic stem cells (HSCs) reside primarily in the bone marrow (BM). This selective location results in part from the unique ability of BM niches to support HSC self-renewal and long-term maintenance. Intense interest in the complex regulation of HSC self-renewal has led to significant progress in understanding the cellular and molecular composition of BM niches (reviewed in Ugarte and Forsberg, 2013). Because osteoblasts are only present in bone, they may provide an environment that helps to regulate the selective location of HSCs to BM. Several lines of evidence support this notion (reviewed in Krause et al., 2013). Recent evidence also points to the vascular endothelium and associated cells as important regulators of HSC maintenance and location (Ding and Morrison, 2013; Ding et al., 2012; Greenbaum et al., 2013; Kunisaki et al., 2013; Méndez-Ferrer et al., 2010; Sacchetti et al., 2007; Sugiyama et al., 2006; Ugarte and Forsberg, 2013), and most HSCs localize near sinusoidal endothelial cells (SECs) (Kiel et al., 2005). Thus, accumulating evidence indicates that vascular structures within the BM are necessary for optimal HSC function.

Another mechanism that is likely involved in specifying HSC location to the BM is regulated trafficking between the BM and vasculature. HSC residence in BM niches is far from static, with circulation in the blood stream occurring under steady-state physiological conditions (Massberg et al., 2007; Wright et al., 2001), between different hematopoietic organs during development, and as an essential requirement for successful hematopoietic transplantation therapies. During trafficking to and from the BM, HSCs have to traverse the vascular endothelium. Differential vascular structures of different organs that either prevent or allow HSC entry likely play important roles in guiding HSCs specifically to the BM. Here, we show that the integrity of the vascular endothelium and its ability to regulate directional HSC trafficking to the BM depend on the single transmembrane cell-surface receptor ROBO4.

We recently reported that ROBO4, expressed by HSCs, promotes HSC localization to BM niches at steady state and upon transplantation (Forsberg et al., 2005, 2010; Smith-Berdan et al., 2011). ROBO4 is a member of the ROBO family of guidance receptors that respond to Slits, secreted proteins that are essential for neuronal development (Brose et al., 1999; Long et al., 2004). ROBO4 was previously identified as an EC-selective protein (Huminiecki et al., 2002; Park et al., 2003) and its support of vascular integrity seems to be particularly important in dynamic situations such as vascular stress, inflammation, and pregnancy (Jones et al., 2008; London et al., 2010; Marlow et al., 2010). ROBO4 was found by our group and others to also be expressed by HSCs, but not hematopoietic progenitor or mature cells (Forsberg et al., 2005, 2010; Ivanova et al., 2002; Shibata et al., 2009; Smith-Berdan et al., 2011). We previously reported that hematopoietic ROBO4 acts as an HSC-selective adhesion molecule that promotes HSC location to BM niches (Smith-Berdan et al., 2011). ROBO4 deletion led to increased numbers of HSCs in the peripheral blood (PB) at steady state and reduced engraftment upon competitive transplantation into wild-type (WT) mice. We also found that CXCR4, a G protein-coupled receptor and well-established regulator of HSC location (Nagasawa et al., 1998; Peled et al., 1999; Zou et al., 1998), was upregulated on ROBO4-deficient HSCs, mitigating the effects of ROBO4 loss. Consequently, ROBO4-deficient HSCs displayed heightened responsiveness to mobilization with the CXCR4 inhibitor AMD3100. Functional differences in the hematopoietic system upon ROBO4 deletion were highly selective for HSCs and did not involve alterations in the number or function of hematopoietic progenitors or mature cells. We also did not detect a defect in cell-cycle status or proliferation of either HSCs or their progeny upon ROBO4 loss or in response to Slits. Similar results were reported independently by others (Goto-Koshino et al., 2012; Shibata et al., 2009). Collectively, these data demonstrated that ROBO4 on HSCs promotes HSC localization to the BM. Here, we report that in addition to ROBO4 expressed by HSCs, endothelial ROBO4 is essential for efficient HSC engraftment. Using a combination of in vitro and in vivo assays, we identify the cellular and molecular mechanisms by which endothelial ROBO4 promotes HSC location to the BM, and reveal strategies for manipulating HSC location.

## Results

### Recipient ROBO4 Promotes HSC Engraftment

**Figure 1:**
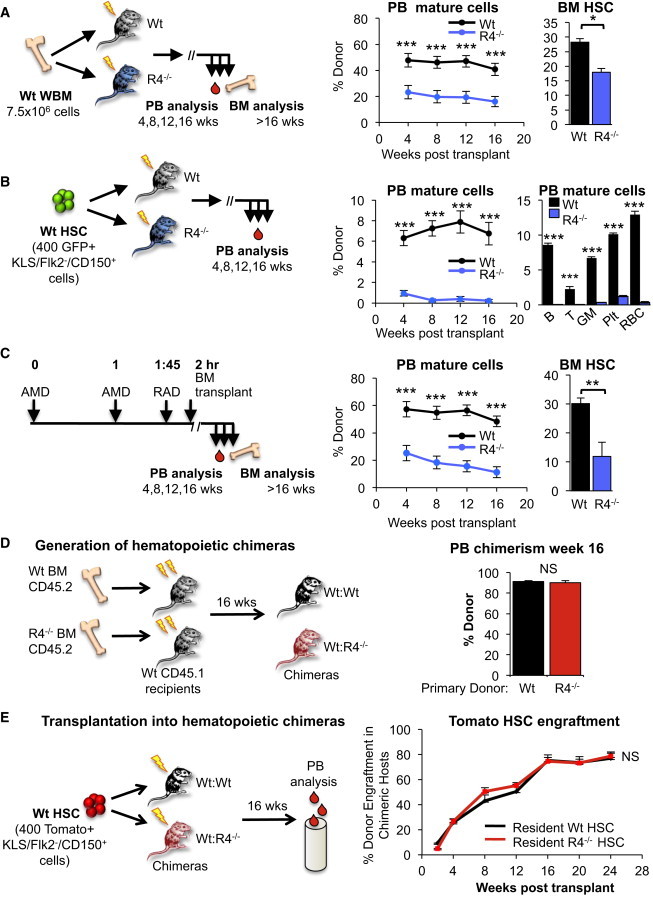
Recipient ROBO4 Is Necessary for Efficient HSC Engraftment (A and B) Engraftment of WT BM cells (A) and purified HSCs (B) is significantly lower in *Robo4*^*−/−*^ compared with WT recipient mice. Middle panels: percent donor contribution to mature cells in the PB over 16 weeks. Right: HSC chimerism in the BM (A) and mature cell chimerism in the PB (B, with donor HSCs from UBC-GFP transgenic mice) at 16 weeks posttransplantation into sublethally irradiated recipients. (A) n = 22 (WT recipients) and 21 (*Robo4*^*−/−*^ recipients) in 4 independent experiments. (B) n = 16 (WT recipients) and 15 (*Robo4*^*−/−*^ recipients) in 3 independent experiments. (C) Engraftment of WT BM cells is significantly lower in *Robo4*^*−/−*^ compared with WT recipient mice preconditioned with both sublethal irradiation and AMD3100. Middle: percent donor contribution in the PB over 16 weeks. Right: HSC chimerism in the BM at 16 weeks posttransplantation. n = 10 recipient mice of each phenotype in 2 independent experiments. (D) Transplantation of WT or *Robo4*^*−/−*^ BM cells (CD45.2) into lethally irradiated WT (CD45.1) recipients to generate mice with either a WT or *Robo4*^*−/−*^ hematopoietic system on a WT background. (E) Mice from (D) with >90% chimerism at 16 weeks posttransplantation were then transplanted with Tomato-expressing WT HSCs into sublethally irradiated recipients and assessed for Tomato-expressing donor cells in PB over time. WT HSC contribution to PB cells was equal in WT recipient mice repopulated by either WT or *Robo4*^*−/−*^ hematopoietic cells. n = 10 mice in 3 independent experiments. R4^−/−^, ROBO4-deficient mice; PB, peripheral blood; BM, bone marrow. HSCs in (B) and (E) were isolated as c-KIT^+^Lin^−^SCA1^+^ (“KLS”) FLK2^−^CD150^+^ BM cells. Error bars represent SEM. ^∗^p < 0.05, ^∗∗^p < 0.005, ^∗∗∗^p < 0.0005. See also Figure S1.

Previous competitive transplantation assays (Smith-Berdan et al., 2011) showed that WT HSCs outcompeted *Robo4*^*−/−*^ HSCs upon transplantation into WT mice. Here, we tested the hypothesis that WT HSCs would also outcompete *Robo4*^*−/−*^ HSCs upon transplantation into *Robo4*^*−/−*^ recipients and therefore engraft with high efficiency in ROBO4-deficient hosts. Surprisingly, we found the opposite: engraftment of WT HSCs was significantly poorer in *Robo4*^*−/−*^ compared with WT recipient mice (Figures 1A and 1B; Figure S1 available online). Similar results were obtained whether HSCs were delivered as unfractionated BM (Figure 1A) or purified HSCs (Figure 1B), and the reconstitution of all mature cell types tested, as well as BM HSCs, was affected (Figures 1A and 1B). Because we previously found that CXCR4 upregulation on *Robo4*^*−/−*^ HSCs attenuates HSC mobilization in response to cytoxan/G-CSF, and mobilization efficiency can be restored with the CXCR4 inhibitor AMD3100 (Smith-Berdan et al., 2011), we tested whether AMD3100 pretreatment of recipient mice could rescue the engraftment deficiency in *Robo4*^*−/−*^ hosts by clearing the host BM niches of resident HSCs. However, similar to what was observed for irradiated recipients, HSC engraftment was more efficient in WT versus *Robo4*^*−/−*^ hosts preconditioned with a combination of irradiation and AMD3100 (Figures 1C and S1). Thus, just as *Robo4*^*−/−*^ HSCs engraft poorly in WT hosts (Smith-Berdan et al., 2011), WT HSCs engraft poorly in *Robo4*^*−/−*^ hosts. We therefore hypothesized that an ROBO4-mediated HSC-HSC interaction between resident and transplanted HSCs facilitates the engraftment of incoming HSCs. We tested this hypothesis in chimeric mice generated by transplantation of WT mice with either WT or *Robo4*^*−/−*^ BM (Figure 1D). Mice with high (>90%) and stable hematopoietic chimerism (Figure 1D) were then transplanted with Tomato-expressing WT HSCs. The engraftment efficiency was indistinguishable between recipient mice that were first repopulated with WT or *Robo4*^*−/−*^ hematopoietic cells (Figure 1E), indicating that the defect in engraftment in *Robo4*^*−/−*^ mice is not due to the absence of ROBO4 on resident HSCs. Thus, we turned our attention to the function of ROBO4 in nonhematopoietic cells.

### Endothelial ROBO4 Promotes Vascular Integrity and HSC Trafficking to the BM

**Figure 2:**
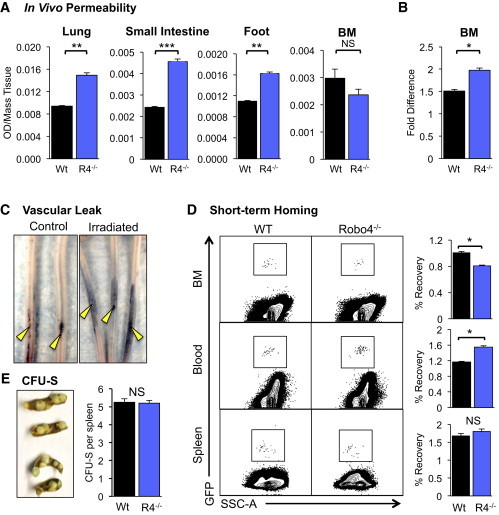
Transplanted HSCs Are Trapped in the Vasculature of ROBO4-Deficient Mice despite Increased Vascular Permeability (A and B) Loss of ROBO4 leads to vascular leak in lung, small intestine, and foot, but not the BM, at steady state (A) and in the BM upon lethal irradiation (B) as measured by leakage of the albumin-binding dye Evans Blue into the indicated tissue 5 min after i.v. injection. n = 9–15 per cohort in 3–5 independent experiments. (C) Sublethal irradiation induces vascular permeability as evidenced by the leakage of Evans Blue dye at the site of tail injection in white (CD1) WT mice. (D) WT HSCs accumulate in the PB of *Robo4*^*−/−*^ compared with WT mice, accompanied by poor HSC recovery from BM, but not spleen, at 3 hr posttransplantation. GFP-expressing KLS (cKIT^+^Lin^−^SCA1^+^) cells were injected into WT or *Robo4*^*−/−*^ recipient mice that had been lethally irradiated 24 hr before. BM, blood, and spleen were analyzed for GFP+ cells 3 hr posttransplantation by flow cytometry and quantified as percent of injected cells recovered from each tissue. n = 3–5 mice per cohort in 5 independent experiments. (E) The ability of transplanted WT HSCs (c-KIT^+^Lin^−^SCA1^+^FLK2^−^CD34^−^ cells) to form spleen colonies (CFU-S_12_) is equal in lethally irradiated WT and *Robo4*^*−/−*^ recipients. n = 16–20 per cohort in 4 independent experiments. Error bars represent SEM. ^∗^p < 0.05, ^∗∗^p < 0.005, ^∗∗∗^p < 0.0005.

In addition to HSCs, ECs also express ROBO4 (Huminiecki et al., 2002; Park et al., 2003). We recently showed that BM ECs, but not other BM stromal cells, express ROBO4 mRNA and cell-surface protein (Smith-Berdan et al., 2012). Therefore, we tested whether endothelial ROBO4 is responsible for the poor HSC engraftment observed in *Robo4*^*−/−*^ mice (Figures 1A–1C). Using intravenously (i.v.) injected Evans Blue in a modified Miles assay (Miles and Miles, 1952), we found that *Robo4*^*−/−*^ mice displayed significantly higher vascular leak in several organs at steady state (Figure 2A). Interestingly, we detected increased vascular leak in the BM of *Robo4*^*−/−*^ mice upon irradiation, but not at steady state (Figure 2B). These results are consistent with previous studies implicating ROBO4 in supporting vascular integrity, in particular under stress conditions (Jones et al., 2008, 2009; Koch et al., 2011), and with compensatory mechanisms attenuating the effects of ROBO4 loss in the BM (see below). We hypothesized that the increased vascular permeability associated with ROBO4 deletion caused transplanted cells to leak out of *Robo4*^*−/−*^ vessels, similar to the leakage of Evans Blue at the tail injection site of irradiated animals (Figure 2C), leading to reduced delivery of HSCs to hematopoietic organs and subsequently lower engraftment levels. However, short-term homing assays revealed that rather than leaking out of vessels, cells transplanted into *Robo4*^*−/−*^ mice were trapped within the vasculature (Figure 2D), with significantly reduced relocation to the BM extravascular space (Figure 2D). Short-term trafficking to the spleen (Figure 2D) and the capability of transplanted HSCs to form spleen colony-forming units (CFU-S) (Figure 2E) did not differ significantly between WT and *Robo4*^*−/−*^ recipients. Thus, ROBO4 deletion selectively affected the ability of WT HSCs to reconstitute the BM, but not the spleen (Figures 1A–1C, 2D, and 2E). Collectively, these results indicate that the poor engraftment in *Robo4*^*−/−*^ recipients is not due to premature HSC leakage out of the vasculature prior to reaching the BM, and that recipient ROBO4 on endothelial, but not hematopoietic, cells affects the engraftment efficiency of transplanted HSCs.

### Endothelial ROBO4 Promotes HSC Translocation across Vascular Barriers

**Figure 3:**
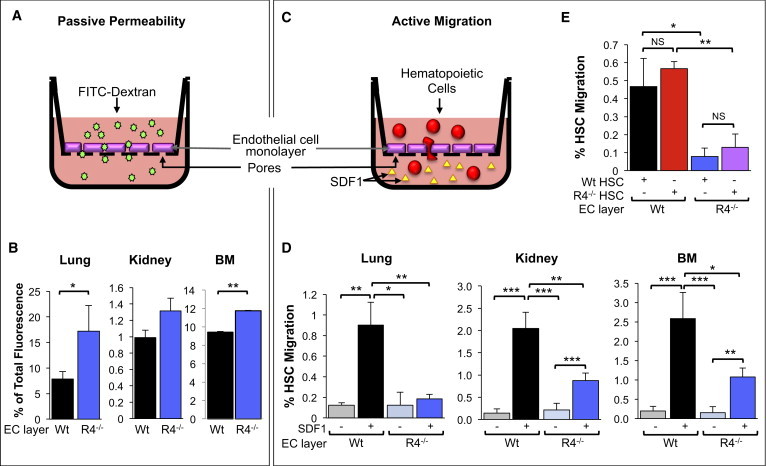
Endothelial ROBO4 Promotes Endothelial Barrier Formation but Is Necessary for Efficient HSC Transendothelial Migration (A and C) Schematic of the strategy to measure passive permeability (A) and active cell migration (C) across WT and *Robo4*^*−/−*^ endothelial cell (EC) layers. (B) Monolayers of *Robo4*^*−/−*^ ECs from lung, kidney, and BM are more permeable to passive diffusion of FITC-dextran compared with WT EC layers. n = 2–5 independent experiments, with 2–3 wells per cohort and experiment. (D) WT HSCs (c-KIT^+^Lin^−^SCA1^+^FLK2^−^CD34^−^ cells) migrate with significantly reduced efficiency across lung, kidney, and BM *Robo4*^*−/−*^ EC layers. n = 2–7 independent experiments, with 2–3 wells per cohort and experiment. (E) HSCs (cKIT^+^Lin^−^SCA1^+^FLK2^−^CD34^−^ cells) from *Robo4*^*−/−*^ mice migrate with the same efficiency as WT HSCs across WT EC layers, and their migration is equally impaired across *Robo4*^*−/−*^ ECs. n = 3–7 independent experiments. Error bars represent SEM. ^∗^p < 0.05, ^∗∗^p < 0.01, ^∗∗∗^p < 0.005. See also Figure S2.

The observation that transplanted cells accumulated in the vasculature (Figure 2D) raised the possibility that endothelial ROBO4 promotes HSC extravasation. To test this directly, we established an in vitro assay system that enabled quantification of both passive and active translocation across endothelial barriers. We isolated primary ECs from the BM, lungs, and kidney of WT and *Robo4*^*−/−*^ mice and grew confluent monolayers on porous transwell inserts. We first tested the integrity of these EC layers by measuring passive diffusion of FITC-labeled dextran, an ∼40 kD macromolecule, to the bottom well (Figure 3A). Although cell phenotype, confluency, and proliferation rates were indistinguishable between WT and *Robo4*^*−/−*^ ECs (Figures S2 and S3B), significantly more FITC-dextran diffused across *Robo4*^*−/−*^ EC layers regardless of the tissue origin of the ECs (Figures 3B and S3C). These results are consistent with the vascular leak in vivo (Figures 2A and 2B) and with previous findings of increased vascular permeability of *Robo4*^*−/−*^ EC layers (Jones et al., 2008). We then tested the ability of HSCs to migrate across lung-, BM-, and kidney-derived EC barriers in response to SDF1 (aka CXCL12), a strong HSC chemoattractant and CXCR4 ligand (Smith-Berdan et al., 2011; Wright et al., 2002; Figure 3C). Remarkably, despite the increased passive permeability of *Robo4*^*−/−*^ relative to WT EC layers, HSC migration across *Robo4*^*−/−*^ EC layers was significantly reduced regardless of the tissue origin of the ECs (Figure 3D). These results show that endothelial ROBO4 facilitates HSC translocation across vascular barriers and that increased passive permeability is not sufficient to overcome the requirement for ROBO4 in promoting active cell translocation. Our findings also identify impaired HSC extravasation as a cause of poor engraftment in *Robo4*^*−/−*^ recipients (Figures 1A–1C).

### Hematopoietic ROBO4 Is Not Necessary for Efficient Transendothelial Migration of HSCs

We previously showed that *Robo4*^*−/−*^ HSCs engraft poorly in WT hosts and attributed this to impaired HSC retention in BM niches, mediated by ROBO4 on HSCs (Smith-Berdan et al., 2011). Because it is possible that ROBO4 on HSCs is also required for efficient extravasation, we tested whether HSC-expressed ROBO4 was important for efficient translocation across EC layers. However, HSCs from *Robo4*^*−/−*^ mice migrated as efficiently as WT HSCs across WT EC barriers and were equally poor at crossing *Robo4*^*−/−*^ EC layers (Figure 3E). These data are consistent with our previous finding that *Robo4*^*−/−*^ HSCs do not display impaired migration toward SDF1 across artificial membranes (Smith-Berdan et al., 2011). Furthermore, the reduced migration across *Robo4*^*−/−*^ EC layers was not specific for HSCs, but also applied to hematopoietic progenitor and mature cells (Figure S3D) that do not express ROBO4 (Smith-Berdan et al., 2011). Because CXCR4 plays a role in vascular development (Tachibana et al., 1998) and *Robo4*^*−/−*^ HSCs upregulate CXCR4 to compensate for the loss of ROBO4 (Smith-Berdan et al., 2011), we tested whether the differential migration across *Robo4*^*−/−*^ EC layers was a result of a differential response to SDF1, the CXCR4 ligand used as the attractant in the transwell assays, on *Robo4*^*−/−*^ versus WT ECs. We did not detect robust levels of CXCR4 on freshly isolated primary BM ECs or cultured EC monolayers by either quantitative RT-PCR or cell-surface stains (Figures S3E and S3F). Neither SDF1 nor other Cxcl genes, SCF, laminins, or PDGFβ were differentially expressed by WT versus *Robo4*^*−/−*^ ECs (Figure S3G). Moreover, SDF1 did not induce detectable differences in EC permeability in vitro (Figure S3H), and IL6-induced migration of B and T cells was less efficient across *Robo4*^*−/−*^ compared with WT ECs (Figure S3I). Thus, the ability of endothelial ROBO4 to promote transendothelial migration is independent of the SDF1/CXCR4 axis.

### ROBO4 Deletion Leads to Alterations in BM Sinusoidal Cell Numbers and Organization

**Figure 4:**
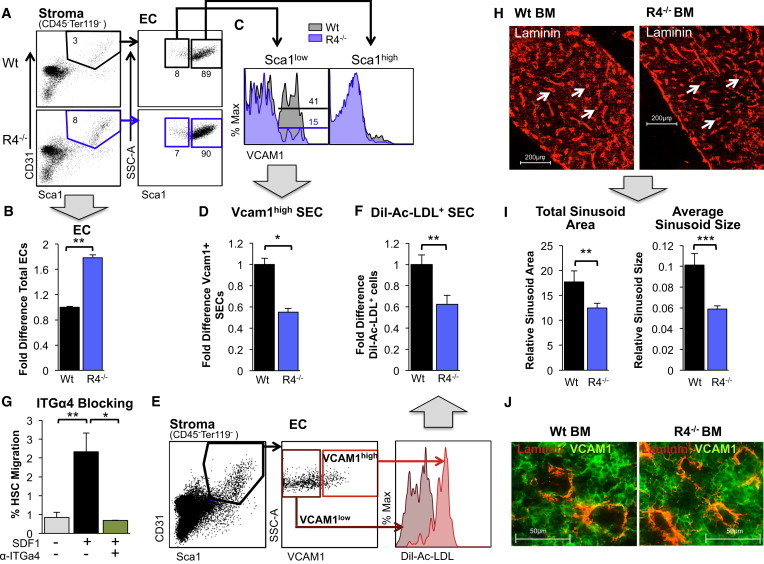
BM Sinusoidal Cell Number and Organization Are Altered in *Robo4*^*−/−*^ Mice (A–D) Flow-cytometry analysis of BM EC populations from WT and *Robo4*^*−/−*^ mice reveals that total EC (CD45^−^TER119^−^CD31^+^SCA1^+^) numbers are increased (B) and SEC (CD45^−^TER119^−^CD31^+^SCA1^low^VCAM1^high^) numbers are decreased (D) in *Robo4*^*−/−*^ compared with WT BM. Broad gray arrows point from representative data to quantification of multiple experiments: (B) represents quantification of ECs as shown in (A), and (D) represents quantification of VCAM1+ SECs as shown in (C). n = 3–5 independent experiments. (E) VCAM1^high^ BM ECs (CD45^−^TER119^−^CD31^+^SCA1^+^) are selectively labeled by i.v. injection of the sinusoidal-cell-selective dye Dil-Ac-LDL, whereas nonsinusoidal (CD45^−^TER119^−^CD31^+^ SCA1^+^VCAM1^low^) ECs are negative for Dil-Ac-LDL. Mice were injected i.v. with Dil-Ac-LDL 4 hr prior to EC harvesting from the BM and flow-cytometry analysis. (F) *Robo4*^*−/−*^ mice have significantly fewer Dil-AC-LDL^+^ BM ECs (CD45^−^TER119^−^CD31^+^SCA1^+^Dil-Ac-LDL^+^ cells) compared with WT mice. Quantification of ECs as shown in (E). n = 7–8 mice in 3 independent experiments. (G) Transendothelial migration of HSCs (c-KIT^+^Lin^−^SCA1^+^FLK2^−^CD34^−^ cells) across WT EC layers in vitro is blocked by preincubation of ECs with an antibody to the VCAM1-binding protein integrin α4. n = 3 independent experiments, with duplicate wells for each experiment and cohort. (H and J) Sinusoids are poorly formed in *Robo4*^*−/−*^ compared with WT mice. BM sections from WT and *Robo4*^*−/−*^ were stained with α-laminin (H) or with α-laminin and α-VCAM1 antibodies (J) and examined for sinusoidal structures by fluorescence microscopy. Note the abundance of sinusoids with a clearly defined lumen in WT BM sections (white arrows in H; laminin^+^/VCAM1^+^ circular structures in J), whereas the sinusoids in *Robo4*^*−/−*^ mice are smaller, with a narrower lumen. (I) The total area and size of the sinusoids from BM sections in (H) were significantly decreased in *Robo4*^*−/−*^ compared with WT BM. n = 3–4 mice for each cohort. Error bars represent SEM. ^∗^p < 0.05, ^∗∗^p < 0.005, ^∗∗∗^p < 0.0005. See also Figures S3 and S4.

To further understand the mechanisms behind endothelial ROBO4-mediated HSC engraftment, we tested the consequences of ROBO4 deletion on the BM vasculature. Quantification of endothelial subpopulations by flow cytometry revealed that the total number of ECs (CD45^−^TER119^−^CD31^+^SCA1^+^ BM cells) was increased in the BM (Figures 4A and 4B), but not in the spleen (Figure S4A), of *Robo4*^*−/−*^ mice. The elevated numbers of BM ECs may prevent detection of increased permeability by Evans Blue leak in *Robo4*^*−/−*^ BM at steady state (Figure 2A). Splenic ECs also expressed lower levels of ROBO4 compared with BM ECs (Figure S3B), indicating that ROBO4 may play less important roles in the splenic vasculature. These data are consistent with the poor trafficking of HSCs selectively to the BM, but not the spleen, in *Robo4*^*−/−*^ mice (Figures 2D and 2E).

We also found that a distinct subpopulation of BM ECs, defined by low levels of SCA1, expressed robust levels of VCAM1 in WT mice, whereas the equivalent VCAM1^high^ population was drastically decreased in *Robo4*^*−/−*^ mice (Figures 4C and 4D). Similarly, VCAM1 mRNA, but not VEGFR2 or VE-Cadherin, was downregulated in ROBO4-deficient ECs (Figure S4C). The SCA1^low^ EC subpopulation expressed high levels of ROBO4, as well as VEGFR2 and VEGFR3 (Figure S4D), and was specifically labeled by i.v. injections of Dil-Ac-LDL, a sinusoidal-cell-specific dye (Kunisaki et al., 2013; Li et al., 2009; Figure 4E). These characteristics are all consistent with an SEC identity (Hooper et al., 2009; Kunisaki et al., 2013; Li et al., 2009). Whether defined by high VCAM1 expression or Dil-Ac-LDL labeling, the number of SECs was significantly reduced in *Robo4*^*−/−*^ BM (Figures 4D and 4F). Consistent with a direct role for VCAM1 in HSC extravasation, blocking antibodies to its binding partner, integrin α4, impaired HSC transendothelial migration in transwell assays (Figure 4G). Equivalent experiments using an antibody reported to block CD31(PECAM1)-mediated leukocyte extravasation (Bogen et al., 1994) had no effect on HSC migration efficiency (Figure S4E). Sinusoidal cells expressing VCAM1 and interacting with integrin α4 thus appear to be important for HSC extravasation into the BM space. Indeed, an examination of BM sections revealed poorly formed sinusoids in *Robo4*^*−/−*^ mice, which was associated with a significant decrease in the total sinusoidal area and average size of individual sinusoids compared with WT BM (Figures 4H–4J and S5). Collectively, these results indicate that ROBO4 loss leads to a poorly developed BM vasculature, characterized by reduced numbers of VCAM1-expressing sinusoidal cells and small, narrow sinusoids. Moreover, the impaired trafficking across ROBO4-deficient endothelium points to BM sinusoids as important conduits for HSC extravasation and subsequent long-term engraftment.

### Vascular Permeability Regulates HSC Mobilization from BM to Blood, but Not HSC Extravasation from Blood to BM

**Figure 5:**
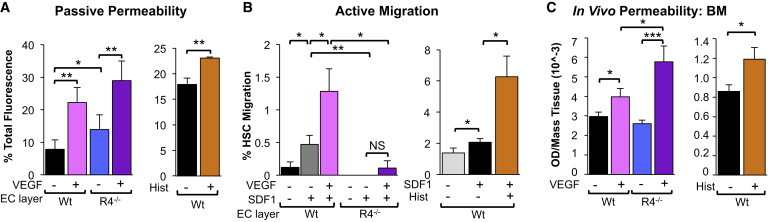
Induced Vascular Permeability Facilitates HSC Transendothelial Migration (A) VEGF and histamine treatment increased the diffusion of FITC-dextran across WT (VEGF and histamine) and *Robo4*^*−/−*^ (VEGF) EC monolayers in vitro. Transwell assays were performed as in Figure 3A except that certain EC layers were treated with VEGF or histamine to induce permeability prior to addition of FITC-dextran to the top wells. n = 3 independent experiments. (B) VEGF- and histamine-induced vascular permeability significantly improved SDF1-induced HSC (c-KIT^+^Lin^low^SCA1^+^FLK2^−^CD34^−^) migration across EC layers in vitro, but VEGF-induced permeability did not rescue the transmigration defect across *Robo4*^*−/−*^ ECs. Transwell assays were performed as outlined in Figure 3C except that specific EC layers were treated with VEGF or histamine to induce permeability prior to addition of hematopoietic cells to the top wells. n = 3 independent experiments. (C) VEGF and histamine treatment induced vascular permeability in the BM of WT (VEGF and histamine) and *Robo4*^*−/−*^ (VEGF) mice. Mice were injected i.v. with Evans Blue and VEGF or histamine, and the amount of Evans Blue leakage into the BM was measured by spectrophotometry. n = 10–19 mice in 4 independent experiments. Error bars represent SEM. ^∗^p < 0.05, ^∗∗^p < 0.005, ^∗∗∗^p < 0.0005. See also Figure S6.

The finding that loss of endothelial ROBO4 impairs transendothelial migration despite causing increased vascular permeability was surprising because we had postulated that increased permeability of vascular barriers would facilitate cellular trafficking. Therefore, we tested whether other means of inducing permeability affected the efficiency of HSC transendothelial migration. We treated EC monolayers with VEGF, the prototype vascular permeability factor (Senger et al., 1993), and confirmed the increased permeability of both WT and *Robo4*^*−/−*^ EC layers by measuring the passive diffusion of FITC-dextran (Figure 5A). In contrast to the impaired translocation of HSCs across the hyperpermeable ROBO4-deficient monolayers (Figures 3B and 3D), VEGF-induced permeability led to significantly improved HSC migration (Figure 5B). Although *Robo4*^*−/−*^ EC monolayers also exhibited increased passive permeability in response to VEGF (Figure 5A), the resulting improvement in cellular translocation was not sufficient to restore transendothelial migration efficiency across *Robo4*^*−/−*^ layers to that observed across WT ECs (Figures 5B, S6A, and S6B). Similar results for both passive permeability and active HSC migration were obtained using histamine (Figures 5A, 5B, S6C, and S6D), a compound that, like VEGF, is capable of rapidly inducing vascular permeability (Ehringer et al., 1996; Woodward and Ledgard, 1986). Thus, the barrier posed by ECs can be reduced by either histamine- or VEGF-mediated permeabilization. Strikingly, the requirement for ROBO4 to promote cell translocation overrides the benefit of the increased vascular permeability caused by either loss of ROBO4 or by VEGF or histamine treatment, resulting in a net decrease in HSC transendothelial migration across *Robo4*^*−/−*^ ECs.

**Figure 6:**
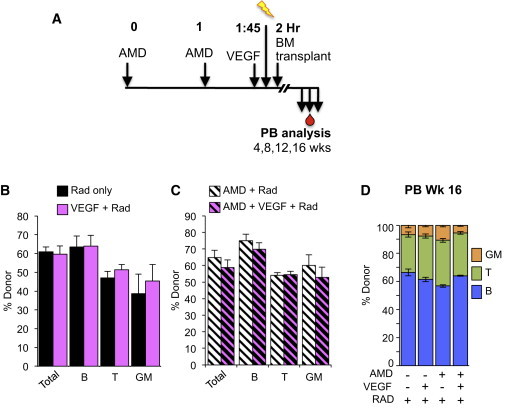
VEGF-Induced Vascular Permeability Does Not Result in Detectable Increases in HSC Engraftment (A) Schematic of recipient mouse preconditioning with AMD3100, VEGF, and/or irradiation prior to BM transplantation. (B) VEGF-induced permeability does not improve engraftment of transplanted HSCs. Donor contribution after transplantation of 7.5 × 10^6^ BM cells from WT (CD45.1) mice was similar whether recipient mice were pretreated with radiation alone (518 rad) or radiation plus VEGF (2 μg/mouse, i.v.). No differences were detectable between pretreatment conditions for up to 16 weeks; data shown were obtained at 8 weeks posttransplantation. n = 3 mice per cohort in each of 3 independent experiments. (C) VEGF-induced permeability does not improve engraftment of transplanted HSCs in recipients preconditioned with radiation and AMD3100. Experiments were performed as in (B) except that recipient mice were also pretreated with AMD3100 (5 mg/kg subcutaneously), as indicated in (A). Two alternative time courses of preconditioning were tested without detectable differences in engraftment. Recipient pretreatment with VEGF alone or in combination with AMD3100 was not sufficient to detect engraftment. n = 3–4 mice per cohort in each of 3 independent experiments. (D) VEGF pretreatment of recipient mice did not alter the lineage distribution of donor cells in recipient mice from (B) and (C). One representative experiment out of three independent experiments is shown. n = 3–4 mice per cohort and experiment. Error bars represent SEM. No comparisons were significantly different. See also Figure S7.

**Figure 7:**
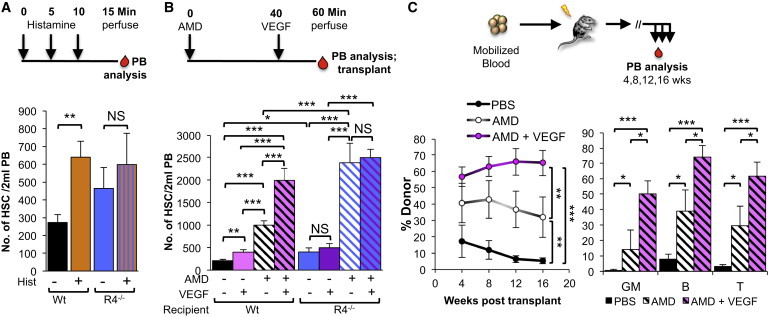
Induced Vascular Permeability Facilitates HSC Mobilization from BM to the Blood Stream (A) Histamine injections induce HSC (c-KIT^+^Lin^low^SCA1^+^CD27^+^FLK2^−^) mobilization to the blood stream in WT, but not *Robo4*^*−/−*^, mice. Three consecutive injections of histamine were followed by perfusion and quantification of HSC numbers in the blood by flow cytometry. n = 3–4 mice per experiment in 3 independent experiments. (B) VEGF, alone or in combination with AMD3100, enhances HSC (c-KIT^+^Lin^low^SCA1^+^CD27^+^FLK2^−^) mobilization to the blood stream in WT, but not Robo4^−/−^, mice. n = 10–18 mice in 3 independent experiments. (C) Long-term, multilineage engraftment of mobilized blood cells from WT mice treated with AMD3100 alone or in combination with VEGF. Blood collected from mice in (E) was transplanted into sublethally irradiated recipient mice, followed by assessment of long-term multilineage reconstitution by flow-cytometry analysis of donor-derived mature cells in the PB of recipient mice. n = 8–9 mice for each cohort in 3 independent experiments. Error bars represent SEM. ^∗^p < 0.05, ^∗∗^p < 0.005, ^∗∗∗^p < 0.0005. See also Figure S7.

Our demonstration here that ROBO4 is necessary for efficient translocation across endothelial barriers seemingly contradicts our previous finding that AMD3100-mediated HSC mobilization from BM to blood is more efficient in *Robo4*^*−/−*^ mice (Smith-Berdan et al., 2011). Therefore, we tested whether VEGF-induced vascular permeability or ROBO4 affected the directionality of HSC movement into and out of the BM space in vivo. As expected, VEGF injections induced robust vascular leak in vivo (Figure 5C). However, VEGF-induced permeability of recipient mice as a pretransplantation conditioning, either by itself or in combination with low-dose irradiation or AMD3100, did not improve short-term or long-term engraftment of transplanted HSCs (Figures 6A–6D). Thus, VEGF-induced permeability did not facilitate HSC translocation from blood to BM. In contrast, VEGF significantly improved HSC mobilization from BM to blood in WT mice (Figure 7B). Remarkably, VEGF significantly enhanced AMD3100-mediated mobilization, leading to extremely rapid and robust increases in phenotypic (Figure 7B) and functional, engraftable HSCs in the PB (Figure 7C), without affecting the relative lineage output of transplanted cells (Figure 7C). Mobilization to the PB was transient, as the numbers of HSCs in the PB, as well as in the BM and spleen, returned to normal levels within 24 hr after drug treatment (Figure S7A). As in our previous report (Smith-Berdan et al., 2011), AMD3100-induced mobilization was more efficient in *Robo4*^*−/−*^ mice (Figure 7B), indicating that endothelial ROBO4 is not necessary for efficient HSC exit from the BM space. Consistent with our in vitro experiments (Figure 5A) and previously published results (Jones et al., 2008), *Robo4*^*−/−*^ mice also responded to VEGF permeabilization (Figure 5C). However, VEGF did not further improve AMD3100-mediated HSC mobilization in *Robo4*^*−/−*^ mice (Figures 7B and S7B), likely due to the already compromised vasculature of *Robo4*^*−/−*^ mice. Like VEGF treatment, histamine injections also led to increased Evans Blue leak (Figure 5C) and to an increase in HSCs in the PB of WT, but not *Robo4*^*−/−*^, mice (Figure 7A), substantiating our conclusion that induced vascular permeability results in HSC mobilization from BM to PB. In contrast to VEGF and histamine, AMD3100 did not induce vascular permeability in vivo (Figure S7B), supporting the notion that AMD3100 acts by directly inhibiting CXCR4-mediated adhesive interactions between hematopoietic cells and the BM environment (Broxmeyer et al., 2005; Smith-Berdan et al., 2011). Hematopoietic progenitors, including MPPs and myeloid progenitors, also mobilized in response to VEGF (Figures S7D–S7F), consistent with VEGF acting on the vasculature as opposed to specific hematopoietic subpopulations. Similar to what was observed for HSCs, VEGF also had a greater effect on progenitor mobilization in WT compared with *Robo4*^*−/−*^ mice (Figures S7D–S7F). Because hematopoietic progenitors do not express ROBO4, the attenuated response to VEGF in *Robo4*^*−/−*^ mice suggests that endothelial ROBO4 helps maintain cells in the BM by preventing vascular leak. Collectively, these results provide compelling evidence that induced vascular permeability enhances HSC mobilization from BM to blood, and that HSC translocation into and out of the BM is regulated by different mechanisms.

## Discussion

### Endothelial ROBO4 Promotes Unidirectional HSC Trafficking across Vessel Walls of the BM

The results presented here show that endothelial ROBO4 is necessary for efficient HSC trafficking from the blood to the BM space. By using a combination of in vitro and in vivo assays, we were able to pinpoint the poor engraftment of WT HSCs in *Robo4*^*−/−*^ mice to defective transendothelial migration. Our finding that HSCs migrate poorly across ROBO4-deficient EC layers despite their increased permeability suggests that the vascular endothelium actively promotes the HSC extravasation process. We found that the number and structure of VCAM1^+^ SECs are compromised in *Robo4*^*−/−*^ BM, and that the VCAM1/ITGA4 interaction is essential for transendothelial migration of HSCs. Together, these findings point to VCAM1^+^ SECs as likely sites for HSC extravasation from blood to BM. As most HSCs reside in close proximity to sinusoidal cells (Kiel et al., 2005), HSCs that exit the blood via sinusoidal structures may take up residence on the BM side of sinusoids. Thus, ROBO4 and VCAM1 on SECs may promote HSC extravasation in the optimal location in the BM.

Intriguingly, the reverse process of HSC escape from the BM to the blood does not require ROBO4. AMD3100-mediated mobilization of progenitor cells was as efficient in the absence of *Robo4*^*−/−*^. Furthermore, AMD3100-mediated HSC mobilization was significantly more efficient in ROBO4^−/−^ mice, consistent with our previous conclusion that ROBO4 on HSCs mediates adhesive interactions with BM niches (Smith-Berdan et al., 2011). Thus, neither ROBO4 nor VCAM1^+^ SECs seem to be essential for efficient HSC mobilization. The alterations in vascular permeability in *Robo4*^*−/−*^ mice led us to also test the role of vascular integrity in HSC retention in the BM. We found that induced vascular permeability, via VEGF and histamine injections, very rapidly mobilized HSCs to the blood stream. This action of VEGF appears to be different from the previously reported VEGF-mediated vascular remodeling and associated HSC relocation that occurs over a period of several days (Hattori et al., 2001). HSC mobilization upon induced vascular permeability indicates that the vasculature acts as a barrier to prevent HSC escape into the blood. Addition of AMD3100, acting on hematopoietic CXCR4, led to increased HSC mobilization compared with ROBO4 loss alone, or VEGF or histamine injection alone. Thus, targeting both the vascular barriers and hematopoietic adhesive forces had additive effects on HSC mobilization, identifying a promising strategy for very rapid and efficient harvesting of HSCs for cell therapies.

Just as ROBO4 and VCAM1 are necessary for efficient HSC extravasation but appear to be dispensable for efficient HSC mobilization, induced vascular permeability improves mobilization but does not appear to enhance HSC extravasation. VEGF preconditioning of recipient mice under conditions that induce vascular permeability did not lead to measureable improvements in HSC engraftment. These observations very clearly separate the mechanisms that regulate HSC trafficking into the BM from those that regulate reentry into the blood. Uncoupling of these mechanisms has been suggested previously (Adams et al., 2009; Méndez-Ferrer and Frenette, 2009). Our results indicate that SECs expressing ROBO4 and VCAM1 promote HSC extravasation, that vascular barriers prevent leakage back to the blood, and that adhesion molecules on HSCs, including ROBO4 and CXCR4, anchor the HSCs to BM niches. In combination, these directional mechanisms result in a net accumulation of HSCs in the BM. Tipping this balance in either direction by modulating these mechanisms, one by one or in combination, would favor HSC location to either the blood for HSC harvesting or to the BM to promote HSC engraftment upon transplantation.

### Vascular Regulation of HSC Location

In addition to affecting directional specificity across vessels, ROBO4 selectively regulates HSC location to the BM. Loss of either endothelial or hematopoietic ROBO4 failed to affect HSC localization to the spleen. Differences in vascular structures throughout the body are likely an important factor in regulating HSC location. Although HSCs can infiltrate different peripheral tissues (Massberg et al., 2007), the vessel walls of most organs likely limit HSC extravasation. In contrast, the splenic vasculature is discontinuous, resulting in unrestricted access of circulating HSCs. HSCs fail to remain in the spleen long term, however, due to the inability of the spleen vasculature to prevent escape to the blood and to support self-renewal. The specialized vascular architecture of the BM, which includes fenestrae and sinusoids, allows HSC entry, long-term engraftment, and regulated reentry to the blood.

Our findings define two distinct mechanisms by which the vascular endothelium promotes HSC location to the BM. By expressing ROBO4, ECs actively promote HSC extravasation from the blood stream to the BM space. In addition, ECs prevent HSCs from traveling in the reverse direction, from the BM to the blood. This vascular barrier formation is supported by ROBO4 and can be manipulated by acute exposure of the endothelium to VEGF or histamine. Our findings reinforce the previously established importance of the vascular endothelium, in particular SECs, in long-term HSC maintenance (Ding and Morrison, 2013; Ding et al., 2012; Greenbaum et al., 2013; Ugarte and Forsberg, 2013) and raise the possibility that secreted niche factors influence HSC location by modulating vascular permeability. Our uncoupling of the mechanisms that regulate the directionality of HSC trafficking and identification of separate molecular targets for each process point to manipulation of vascular integrity as a strategy for improving both HSC mobilization and engraftment in clinical therapy.

## Experimental Procedures

### Mice

Mice were maintained by the University of California, Santa Cruz, animal facility according to approved protocols. *Robo4*^*−/−*^ mice on the C57Bl6 background were described previously (Jones et al., 2008; London et al., 2010; Marlow et al., 2010). UBC-GFP and mTmG transgenic mice (both from JAX) were described previously (Boyer et al., 2011, 2012; Muzumdar et al., 2007; Schaefer et al., 2001).

### Transplantation Assays

Long-term reconstitution, short-term homing, and CFU-S assays were performed similarly to previous protocols (Boyer et al., 2011; Forsberg et al., 2006; Smith-Berdan et al., 2011) and are described in detail in Supplemental Experimental Procedures.

### EC Isolation and Culturing

ECs were isolated from lungs, kidney, or BM from WT or *Robo4*^*−/−*^ mice and treated with collagenase as previously described (Dong et al., 1997; Jones et al., 2008; Sobczak et al., 2010). Freshly isolated cells were used for gene and protein expression analyses. For transwell assays, magnetic-bead-enriched cells were cultured for up to four passages under conditions that promote EC growth (Sobczak et al., 2010).

### Transendothelial Migration Assays

Primary ECs were seeded onto 0.5% gelatin-treated transwell inserts and grown to confluency. BM cells (lineage depleted by magnetic selection when appropriate) from WT or *Robo4*^*−/−*^ mice were preincubated at 37°C for 1 hr and then placed in the upper chamber of a transwell insert (5 μm pore size). The bottom wells contained SDF1 (100 ng/ml) or IL-6 (100 ng/ml) (Weissenbach et al., 2004) as indicated. Cells were allowed to migrate for 2 hr at 37°C before harvesting and analysis by flow cytometry as described previously (Smith-Berdan et al., 2011). In some cases, permeability was induced by exposing starved cells to 2.4 nM rhVEGF-165 for 3.5 hr or to 32 μM histamine for 45 min prior to migration. For blocking assays, ECs were pretreated with anti-ITGα4 (clone PS/2) (Bowden et al., 2002) or anti-CD31 (clone 2h8) (Bogen et al., 1994) antibodies at 10 μg/ml for 30 min prior to cell migration toward SDF1.

### Vascular Permeability Assays

A modified Miles assay was utilized to assess in vivo vascular permeability (Miles and Miles, 1952). Mice were injected i.v. with Evans Blue (50 mg/kg) and then euthanized by isoflurane inhalation. Vascular leak was determined by isolating tissues at 5–10 min postinjection and measuring Evans Blue absorbance, expressed as OD650/tissue mass. For induced permeability assays, VEGF (2 μg/mouse) was injected once i.v., followed by Evans Blue dye 5 min later. The dye was allowed to leak into the tissues for an additional 15 min prior to tissue harvest, whereas histamine (100 μg/mouse) was injected i.v. three times 5 min apart, followed by Evans Blue dye i.v. 5 min later and 5 min prior to tissue harvest. For radiation permeability assays, mice were treated with a lethal dose of radiation 3 days prior to the Miles assay.

### Immunohistochemistry

Bones were embedded into OCT cryopreservation media on an ethanol/dry ice slurry immediately after dissection from WT or *Robo4*^*−/−*^ mice and stored at −80°C. BM sections (7 μm thick) were cut with a tungsten blade and fixed with acetone at −20°C for 10 min or with 4% paraformaldehyde at 4°C for 20 min. Sections were blocked with 10% goat serum prior to overnight antibody staining at 4°C, followed by incubation for 1 hr with fluorescently conjugated secondary antibodies. Samples were imaged with a Keyance microscope.

### Mobilization

Mice were injected with either histamine (5 mg/kg i.v.) or AMD3100 (5 mg/kg subcutaneously) and/or rhVEGF-165 (2 μg/mouse, i.v.) as indicated. Total blood was isolated by perfusion with PBS/20 mM EDTA and processed for cell counts and flow-cytometry analysis to determine the number and frequency of each cell population as described previously (Smith-Berdan et al., 2011). Reconstitution assays on mobilized blood were performed by transplanting one-half of the blood mouse equivalent into a lethally irradiated host (1,024 rads). Recipient mice were bled at the indicated intervals after transplantation via the tail vein, and PB was analyzed for donor chimerism as described above and previously (Beaudin et al., 2014; Boyer et al., 2011, 2012; Forsberg et al., 2006; Ooi et al., 2009; Smith-Berdan et al., 2011).

### Statistics

Statistically significant differences for all comparisons were calculated using two-tailed t tests unless stated otherwise. One-sided t tests were used for comparison with zero values (Figure 5B).

## Supplemental Information

Document S1. Supplemental Experimental Procedures and Figures S1–S7

Document S2. Article plus Supplemental Information
