# Supplementary material for: ROBO4-Mediated Vascular Integrity Regulates the Directionality of Hematopoietic Stem Cell Trafficking
Source: Stem Cell Reports. 2015 Jan 29;4(2):255–68. doi: 10.1016/j.stemcr.2014.12.013 (PMC4325232; doi:10.1016/j.stemcr.2014.12.013)
Supplement: Document S1. Supplemental Experimental Procedures and Figures S1–S7 [file mmc1.pdf]

Stem Cell Reports, Volume *4*

Supplemental Information

ROBO4-Mediated Vascular Integrity Regulates the Directionality of Hematopoietic Stem Cell Trafficking

Stephanie Smith-Berdan, Andrew Nguyen, Matthew A. Hong, and E. Camilla Forsberg

Supplemental Figure 1

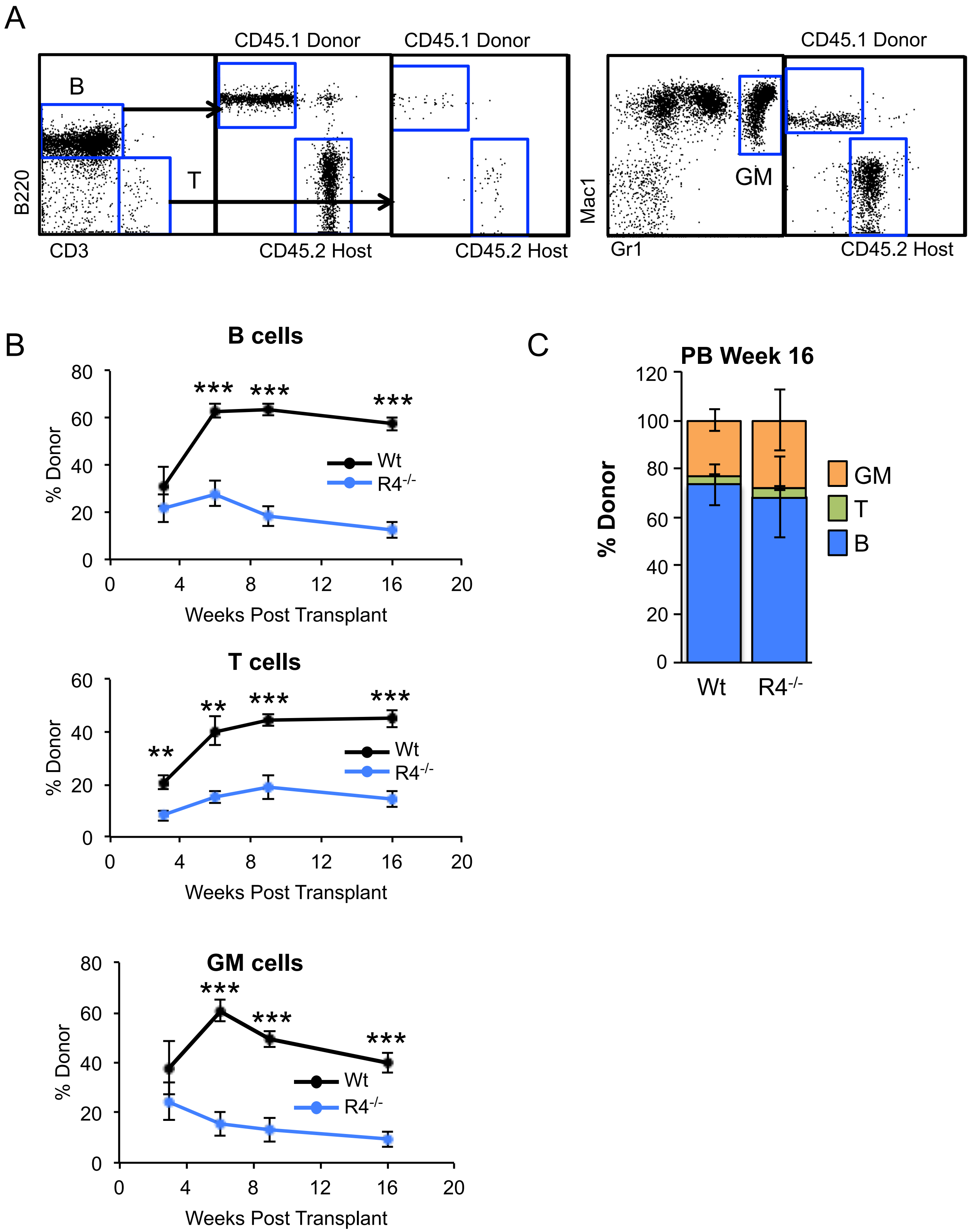

Supplemental Figure 1. Robo4 on recipient mice regulates engraftment of wt HSCs.

(A,B) Quantification and representative flow cytometry plots of donor contribution to B ($B220^{+}CD3^{-}Mac1^{-}Gr1^{-}Ter119^{-}$), T ($CD3^{+}B220^{-}Mac1^{-}Gr1^{-}Ter119^{-}$) and granulocyte/macrophage (GM; $Gr1^{+}Mac1^{+}B220^{-}CD3^{-}Ter119^{-}$) cells in the peripheral blood of sublethally irradiated recipient mice in Figure 1.

(C) Lineage distribution of wt donor cells was not different in wt or $Robo4^{-/-}$ recipient mice. Data shown is from recipient mice preconditioned with both AMD3100 and sublethal irradiation, then transplanted with unfractionated BM cells from wt mice, as outlined in Figure 1C. n = 22 Wt mice and n = 21 $Robo4^{-/-}$ mice.

$R4^{-/-}$ denotes Robo4-deficient mice; PB, peripheral blood. Error bars represent standard error of the mean (SEM). *$p < 0.05$, **$p < 0.001$, ***$p < 0.0001$.

Supplemental Figure 2

Passage 0

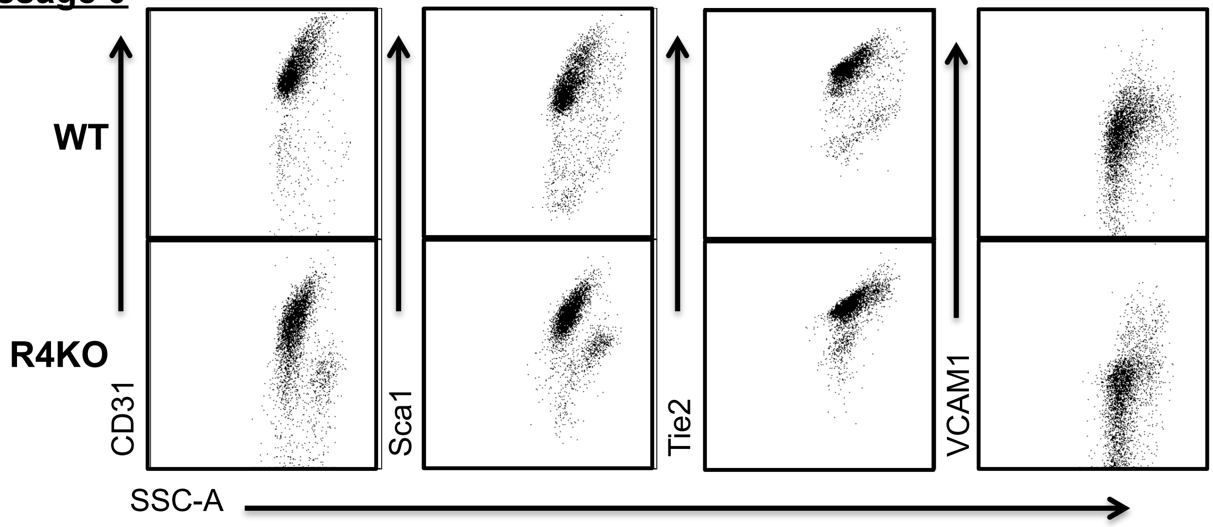

Passage 1

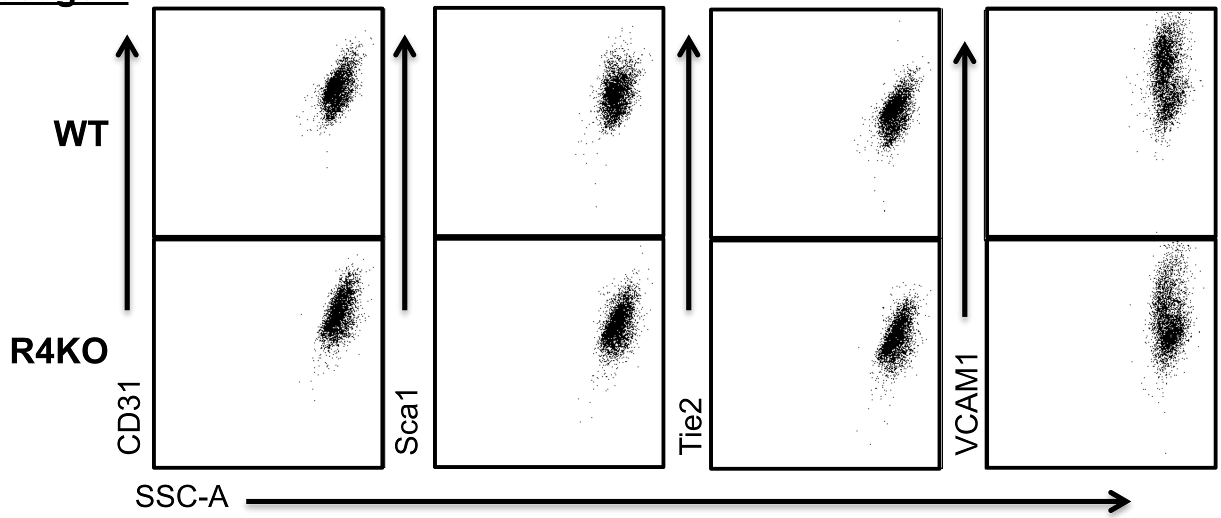

Passage 2

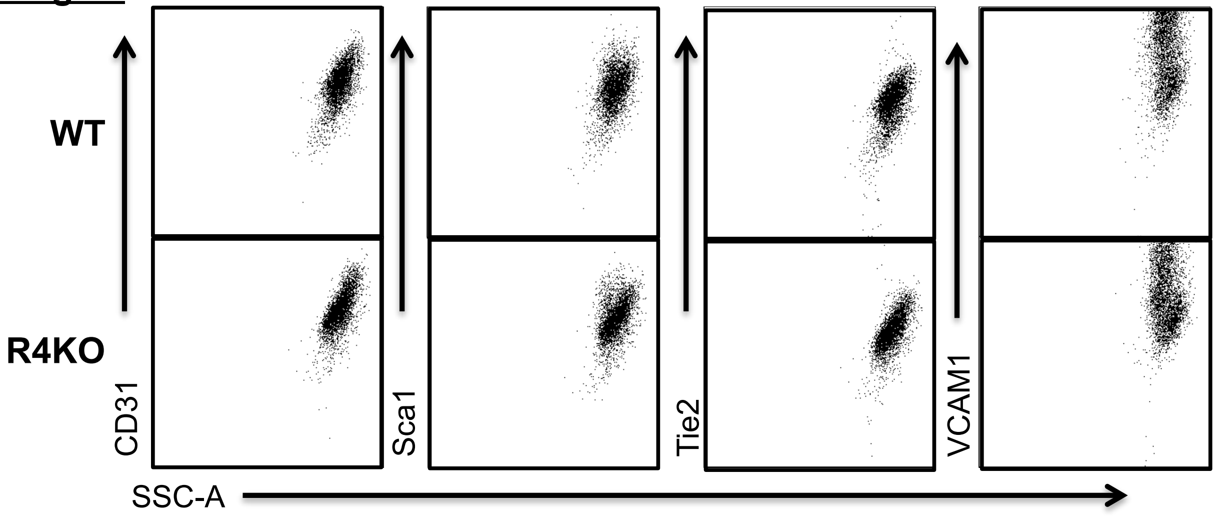

Supplemental Figure 2. Cell surface phenotype of endothelial cells cultured for transwell assays.

The vast majority of CD31-enriched bone marrow stromal cells isolated from wt and Robo4$^{-/-}$ mice express the cell surface proteins CD31, Sca1 and Tie2 (Tek). Freshly isolated cells express low to intermediate levels of Vcam1, whereas the majority of cells beyond 1-2 passages expressed robust levels of Vcam1. Plots show representative flow cytometry results of cells freshly isolated (passage 0) from mouse bone marrow, or the same cells grown in culture under conditions promoting endothelial cell growth (passage 1 and 2) using the indicated antibodies.

Supplemental Figure 3

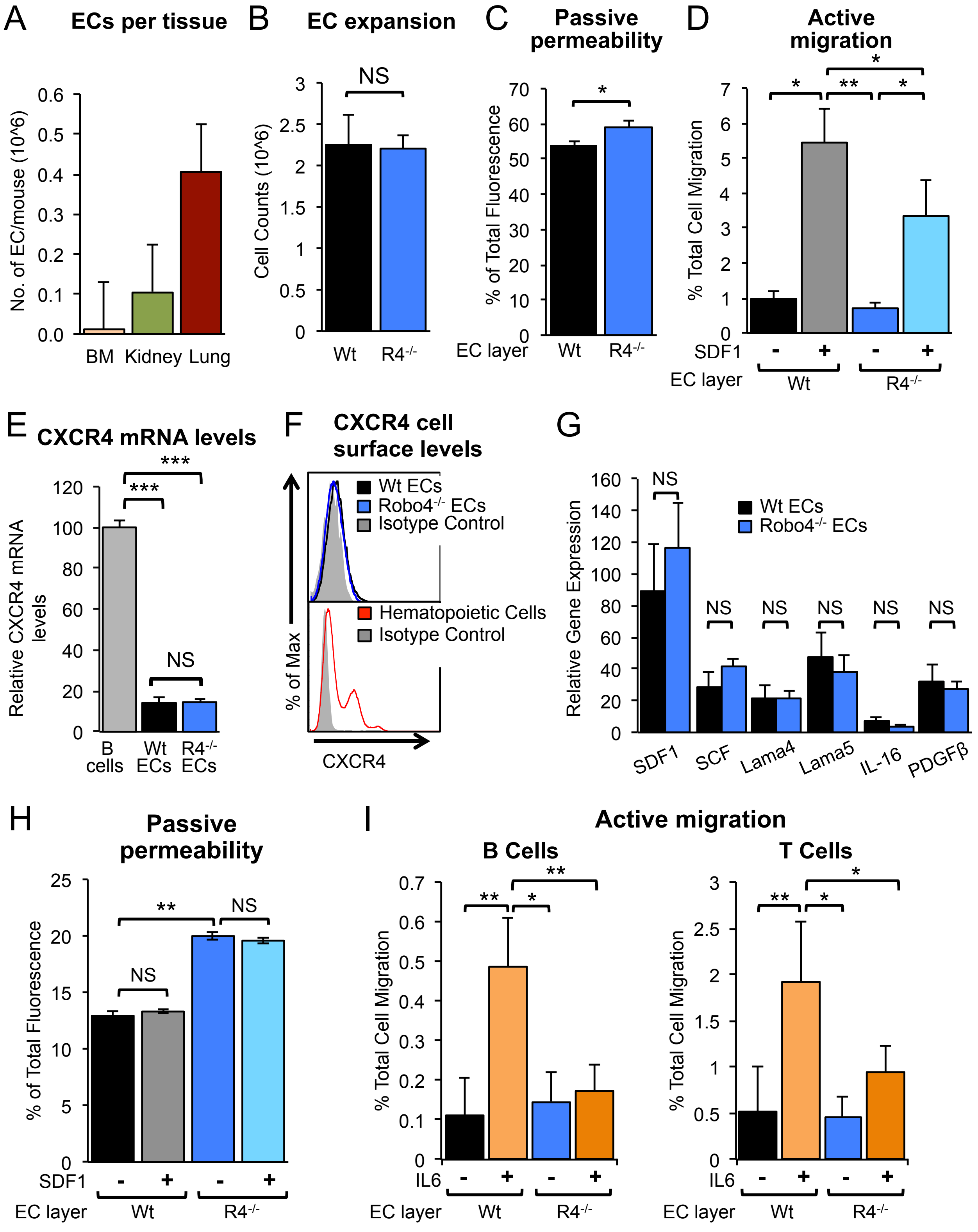

Supplemental Figure 3. The ability of endothelial Robo4 to promote transendothelial migration of hematopoietic cells is independent of SDF1 and CXCR4.

(A) The numbers of endothelial cells recovered from lung is far greater than from kidney (~4-fold) and BM (~40-fold). Primary cells were isolated by collagenase I treatment of the indicated tissue, as described previously (Smith-Berdan et al., 2012). Anti-CD45 and/or anti-CD31 magnetic beads were used for negative and positive selection, respectively, to obtain cells highly enriched for endothelial cells. Cells from each tissue were counted. n = 10 mice in 3 independent experiments.

(B) The numbers of wt and Robo4$^{-/-}$ cells in confluent endothelial layers were equal. Primary endothelial cells (ECs) were isolated from wt or Robo4$^{-/-}$ mice using anti-CD31 magnetic beads and cultured under conditions optimized for endothelial cell growth. Cells were counted 5-7 days after initial isolation or 5-7 days after the first split. n = 8 experiments.

(C) Robo4$^{-/-}$ EC layers are more permeable to macromolecule diffusion than wt EC layers, as measured by Cascade Blue-dextran (molecular weight ~10kDa) accumulation in bottom wells of transwell plates. Experiments were performed as outlined in Figure 3A. n = 4 experiments, *p<0.05.

(D) The efficiency of transendothelial migration of lineage-depleted hematopoietic BM cells is reduced across Robo4$^{-/-}$ EC layers compared to wt EC layers. Experiments were performed as outlined in Figure 3C. The recovery of total cells, regardless of phenotype, in the bottom wells were quantified and expressed as a fraction of total cells added to top wells, as described previously (Smith-Berdan et al., 2011). n = 7 experiments, *p<0.05, **p<0.001.

(E) Endothelial cells isolated from wt and Robo4$^{-/-}$ BM do not express robust mRNA levels of CXCR4. qRT-PCR of CD45$^-$Ter119$^-$CD31$^+$Sca1$^+$ BM endothelial cells isolated as in (Smith-Berdan et al., 2012). n = 4 - 6 independent experiments, each performed in triplicate. ***p < 0.0001

(F) ECs isolated from wt and Robo4$^{-/-}$ mice do not display detectable cell surface levels of CXCR4 (left panel). Anti-CD31 magnetic beads were used to enrich for CD31-expressing BM cells. ECs were further identified by flow cytometry as CD45$^-$Ter119$^-$CD31$^+$ cells. A subset of coisolated hematopoietic cells, defined as CD45$^+$ by flow cytometry, expressed readily detectable CXCR4 cell surface levels (right panel) in the same experiments. n = 2 experiments.

(G) Wt and Robo4$^{-/-}$ ECs express similar levels of SDF1 (Cxcl12), SCF (KitL), Laminin 4 and 5, IL-16 and PDGFβ. mRNA levels were assessed in ECs isolated from mouse BM by flow cytometry as CD45^{-}Ter119^{-}CD31^{+}Sca1^{+} cells and displayed relative to β-actin (arbitrarily assigned a value of 100). n = 3 independent experiments.

(H) SDF1 does not induce detectable changes in the permeability of either wt or Robo4$^{-/-}$ EC layers. EC layers were incubated with SDF1 (100 ng/ml) for 2 hours, then tested for permeability by measuring FITC-dextran diffusion to the bottom chamber. n = 3 experiments, **$p<0.01$.

(I) IL6-induced transendothelial migration of B (left) and T (right) cells is reduced across Robo4$^{-/-}$ compared to wt EC layers. Experiments were performed as outlined in Figure 3C, except IL6 was used as the attractant in the bottom wells instead of SDF1. n=3 independent experiments in duplicate. *$p<0.05$, **$p<0.01$.

Error bars represent SEM.

Supplemental Figure 4

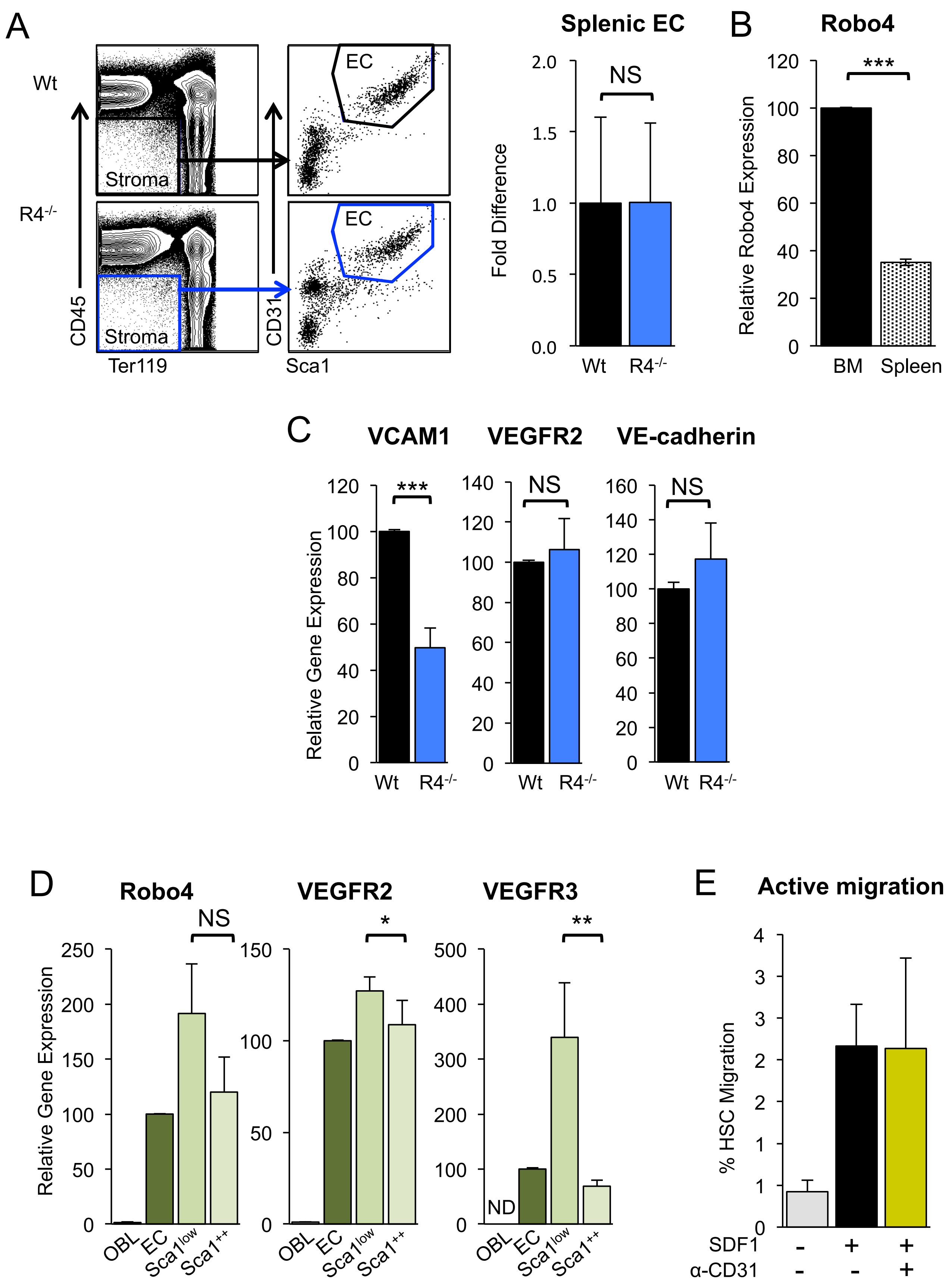

Supplemental Figure 4. Characterization of BM and spleen endothelial cells in wt and Robo4$^{-/-}$ mice.

(A) The numbers of splenic endothelial cells are not altered upon loss of Robo4. Cells were isolated from spleens of wt and Robo4$^{-/-}$ mice by collagenase digestion, then analyzed by flow cytometry with the indicated antibodies. The numbers of total endothelial cells (CD45^{-}Ter119^{-}CD31^{+}Sca1^{+}) from wt and Robo4$^{-/-}$ spleens were enumerated. n = 3 independent experiments with 3 mice per experiment and cohort.

(B) Robo4 is expressed at significantly lower levels in splenic versus BM endothelial cells. Quantitative RT-PCR of Robo4 mRNA in CD45^{-}Ter119^{-}CD31^{+}Sca1^{+} cells from BM or spleen from wt mice. n = 3-4 individual experiments.

(C) Vcam1, but not VEGFR2 or VE-cadherin, expression is downregulated in Robo4$^{-/-}$ compared to wt BM endothelial cells. Quantitative RT-PCR of Vcam1, VEGFR2, and VE-cadherin mRNA levels in BM endothelial cells (CD45^{-}Ter119^{-}CD31^{+}Sca1^{+}) isolated from wt and Robo4$^{-/-}$ mice. n = 4-9 independent experiments.

(D) Relative expression of Robo4, VEGFR2, and VEGFR3 in different BM cell populations. Quantitative RT-PCR of Robo4, VEGFR2 and VEGFR3 mRNA levels in osteoblast lineage BM cells (CD45^{-}Ter119^{-}CD31^{-}CD51^{+}Sca1^{-} BM cells), total endothelial cells (CD45^{-}Ter119^{-}CD31^{+}Sca1^{+} BM cells) and endothelial cell subpopulations (sinusoidal CD45^{-}Ter119^{-}CD31^{+}Tie2^{+}Sca1^{low} and non-sinusoidal CD45^{-}Ter119^{-}CD31^{+} Tie2^{+}Sca1^{high} BM cells). n = 3-4 individual experiments.

(E) Addition of blocking antibodies to CD31 (aka PECAM1) does not impair HSC transendothelial migration in transwell assays. Monolayers of endothelial cells from wt mice were preincubated with anti-CD31 monoclonal antibodies prior to addition of hematopoietic cells to the upper chamber, similar to the outline in Figure 3C. The number of HSCs that migrated towards an SDF1 gradient in the bottom chamber was quantified by flow cytometry after a 2-hour migration period at 37C. n = 2 individual experiments with each condition performed in duplicate.

Error bars represent SEM. *p<0.05, **p<0.005, **p<0.0005.

Supplemental Figure 5

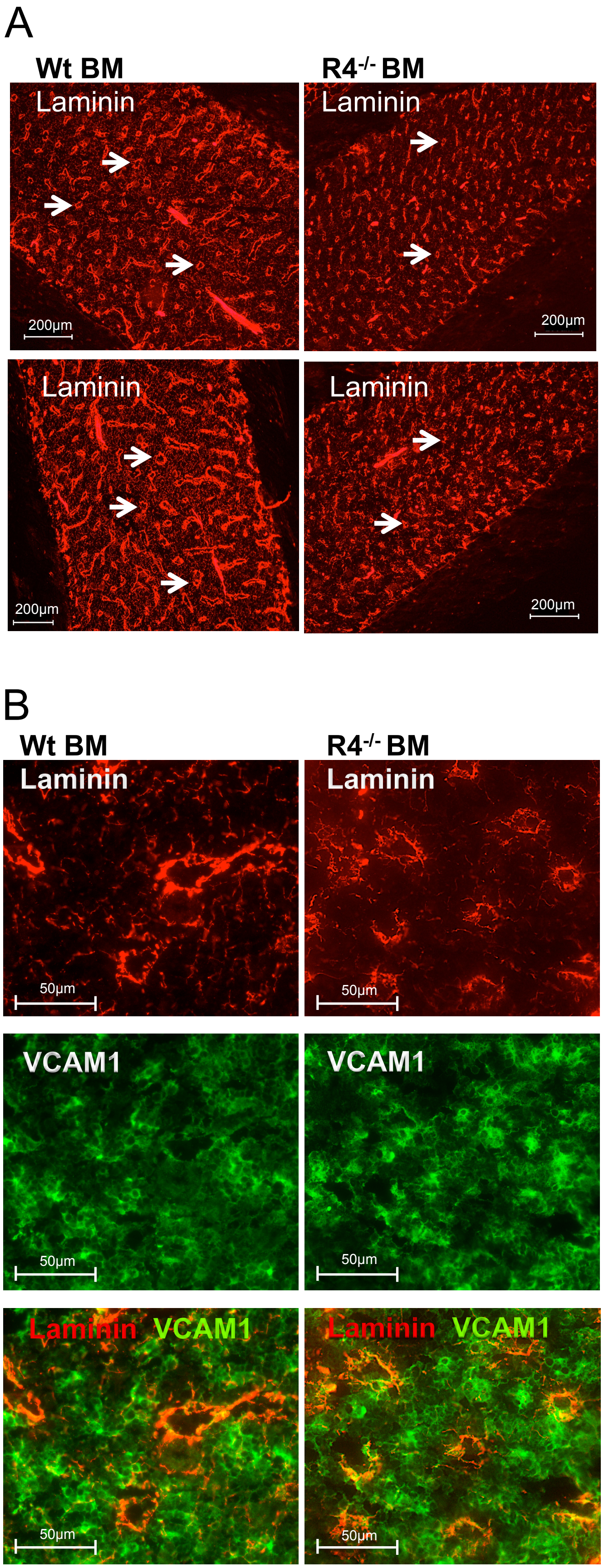

Supplemental Figure 5. Robo4 deletion results in poorly formed BM sinusoids.

(A) BM sections from wt and Robo4$^{-/-}$ mice were stained with α-laminin antibodies, inspected by microscopy and quantified as shown in Figure 4I. Shown here are additional representative images of Figure 4h. Note the abundance of rounded sinusoids (white arrows) in wt BM sections, whereas sinusoids in Robo4$^{-/-}$ BM sections appeared small and narrow.

(B) Sinusoids in wt mice were visibly larger than sinusoids in Robo4$^{-/-}$ mice. BM sections from wt and Robo4$^{-/-}$ mice were stained with α-laminin and α-Vcam1 antibodies. Shown here are additional representative images of Figure 4J, with each color channel shown separately and combined. Note that many hematopoietic cells express Vcam1, leading to extensive green fluorescence.

Supplemental Figure 6

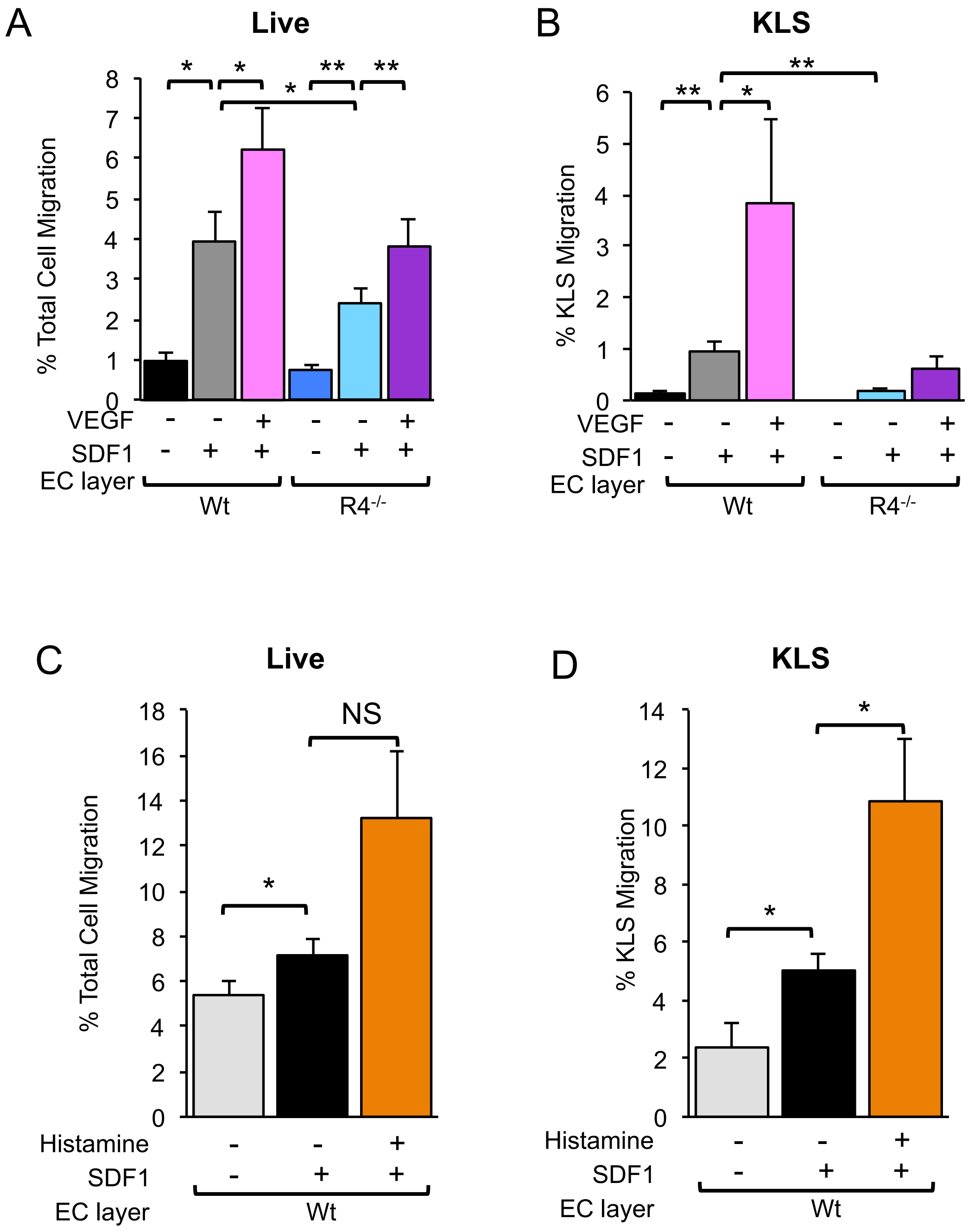

Supplemental Figure 6. Increased vascular permeability improves hematopoietic cell migration across endothelial cell monolayers.

(A,B) Transendothelial migration of lineage-depleted BM cells (A) or hematopoietic stem and progenitor cells ($kit^{+}Lin^{-}Sca1^{+}$ BM cells) (A) across both wt and $Robo4^{-/-}$ EC layers is improved by VEGF-induced permeabilization. These transwell experiments were performed as outlined in Figure 3C, except VEGF was used as a pretreatment of the indicated wells. n = 7, *p<0.05, **p<0.01.

(C,D) Transendothelial migration of lineage-depleted BM cells (C) or hematopoietic stem and progenitor cells ($kit^{+}Lin^{-}Sca1^{+}$ BM cells) (D) across wt endothelial monolayers is improved by histamine-induced permeabilization. Experiments were performed as above and as outlined in Figure 3C, except histamine was used as a pretreatment of the indicated wells. n = 3 individual experiments with each condition performed in duplicate, *p<0.05.

Error bars represent SEM.

Supplemental Figure 7

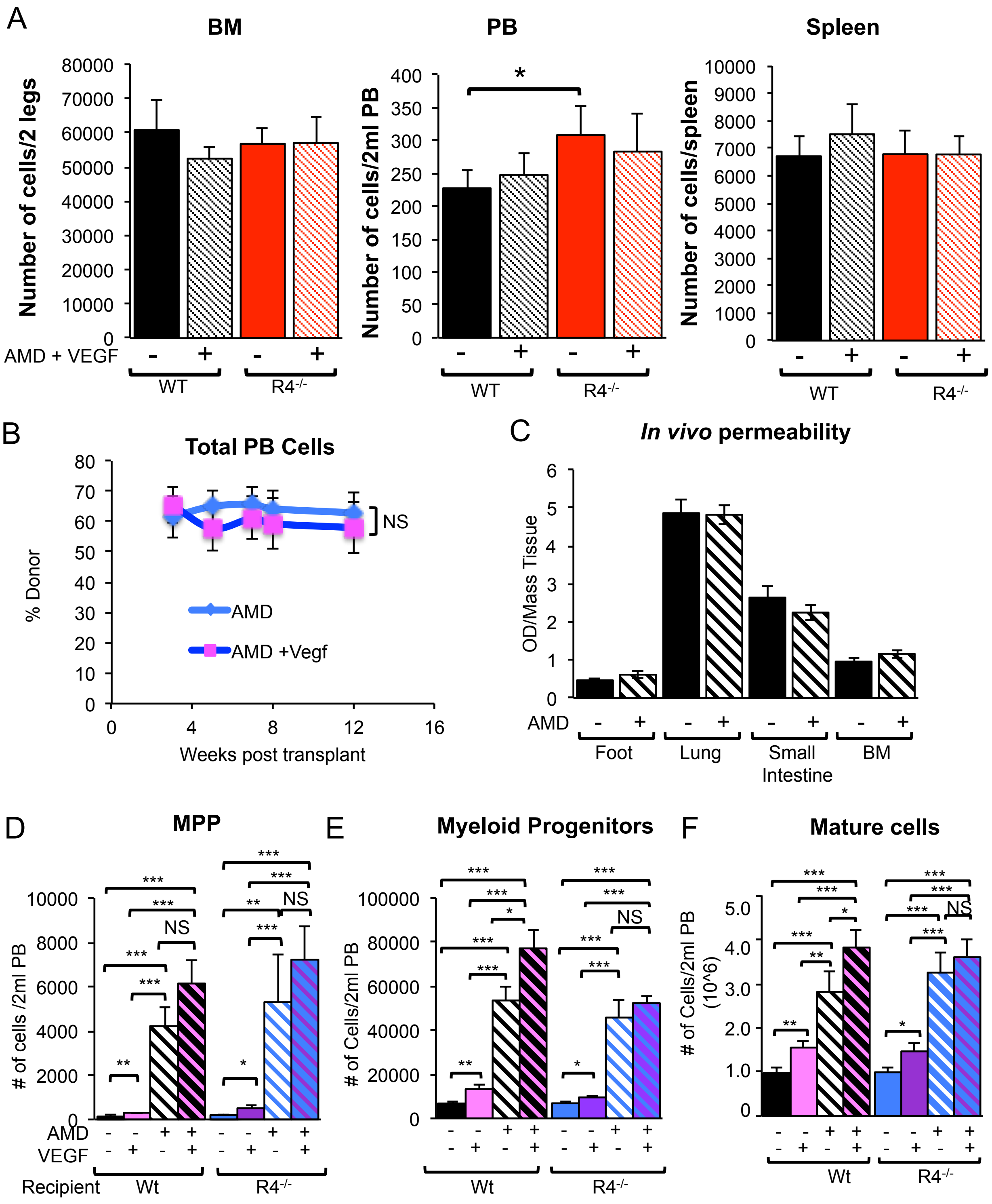

Supplemental Figure 7. VEGF induces mobilization of hematopoietic progenitors from BM to blood.

(A) AMD3100+VEGF-mediated HSC mobilization is transient. Twenty-four hours after mobilization of wt and Robo4$^{-/-}$ mice with a combination of AMD3100 and VEGF, the numbers of HSCs in the BM, PB and spleen were similar to that of untreated control mice. The mice were treated with AMD3100 and VEGF according to the schedule in Figure 7B, except analysis for HSCs was performed 24 hours after AMD3100 injection. HSCs were defined as cKit^{+}Lin^{-}Sca-1^{+}Flk2^{-} cells in the BM and spleen, and also gated on CD27^{+} cells in the PB. n = 3 mice per cohort in 3 independent experiments.

(B) AMD3100+VEGF-mediated HSC mobilization does not result in higher numbers of reconstituting cells in the PB in Robo4$^{-/-}$ mice compared to AMD3100 treatment alone. Robo4$^{-/-}$ mice were treated with either AMD3100 alone or with a combination of AMD3100 and VEGF according to the schedule in Figure 7B. Transplantation of PB and analysis of reconstitution in wt recipients was performed as in Figure 7C. n = 3-4 mice per cohort in each of 2 independent experiments.

(C) AMD3100 does not induce detectable changes in vascular permeability *in vivo*. Mice were treated AMD3100 (subcutaneous injection of 5 mg/kg) 1 hour prior to Evans Blue injection (intravenously; 50 mg/kg). Tissue leak was assessed 5 minutes after Evans Blue injection by optical density measurement of blue dye at 650 nm in surrounding tissue, then divided by the mass of the tissue (Miles and Miles, 1952). n = 3 mice per cohort in 3 independent experiments.

(D-F) VEGF-induced vascular permeability improves mobilization of (D) multipotent progenitor cells (cKit^{+}Lin^{-}Sca-1^{+}Flk2^{+}CD27^{+} cells), (E) myeloid progenitor cells (cKit^{+}Lin^{-}Sca-1^{-} cells), and (F) mature hematopoietic cells (lineage^{+} cells) from BM to blood. Mice were treated with VEGF and/or AMD3100 as outlined in Figure 5e. Numbers of cells of each phenotype in the blood was quantified by flow cytometry. n = 10-18 mice in 3 independent experiments. *p < 0.05, **p < 0.005, ***p < 0.0005. Error bars represent SEM.

SUPPLEMENTAL EXPERIMENTAL PROCEDURES

Flow Cytometry Method. Hematopoietic stem cells (cKit+/Lin-/Sca1+/Flk2-/CD34-) or (cKit+/Lin-/Sca1+/Flk2-/SLAM+), endothelial cells (CD45-/Ter119-/CD31+/Sca1+), VCAM1+ ECs (CD45-/Ter119-/CD31+/Sca1low/Tie2+/VCAM1+), and VCAM1^{low} ECs (CD45-/Ter119-/CD31+/Sca1++/Tie2+/VCAM1low) were prospectively isolated using a FACS ARIA II (BD Biosciences). Similar populations and those previously described in the methods were analyzed by either a FACS ARIA II or an LSRII flow cytometer (BD Biosciences). All hematopoietic stem cells were double sorted on low pressure with a 100um nozzle and the endothelial cell populations were double sorted using either a 100um or 130um nozzle on low pressure. The antibodies utilized for HSC isolation were: cKit-PECy7 (Biolegend, Cat # 105814), Sca1-PB (Biolegend, Cat # 122520), Flk2-Biotin (BioLegend, Cat # 135308), streptavidin-Qdot605 (Invitrogen, Cat # Q10101MP), Slam (CD150) – PE (BioLegend, Cat # 115904), Flk2-APC (BioLegend, Cat # 135310), CD34-FITC (eBioscience, Cat # 11-0341-85), Purified Lineage: CD3 (BioLegend, Cat # 100331), CD4 (BioLegend, Cat # 100402), CD5 (BioLegend, Cat # 100602), CD8 (BioLegend, Cat # 100702), B220 (BioLegend, Cat # 103202), Ter119 (BioLegend, Cat # 116202), Mac-1 (BioLegend, Cat # 101202), and Gr-1 (BioLegend, Cat # 108402), Cy5-R-PE Goat anti-Rat IgG (H+L) (Invitrogen, Cat # A10691). The antibodies utilized for EC isolation were: CD31-Alexa 488 (BioLegend, Cat # 102414), CD31-APC (BioLegend, Cat # 102410), Sca1-PB (Biolegend, Cat # 122520), CD45.2-Alexa700 (BioLegend, Cat # 109822), Ter119-PECy5 (BioLegend, Cat # 116210), Tie2-PE (BioLegend, Cat # 124008), and VCAM1-PECy7 (BioLegend, Cat # 105720).

Transplantation assays. Unfractionated BM cells, or HSCs (cKit^{+}/Lin^{-}/Sca1^{+}/Flk2^{-}/CD150^{+} BM cells) from C57BL/6 mice (with fluorescent transgenes, UBC-GFP or mTmG (Boyer et al., 2012; Boyer et al., 2011; Muzumdar et al., 2007; Schaefer et al., 2001)) isolated by two rounds of FACS, were administered IV into congenic hosts. Recipient radiation was delivered as a single, sublethal dose (518 rads) or a split lethal dose (1040 rads) administered 3 hours apart using a Faxitron CP160 X-ray instrument. AMD3100 (5 mg/kg) was administered to recipients prior to cell transplantation according

to the schedule outlined in Figures 1C and 6A. Recipient mice were bled at the indicated intervals post transplantation via the tail vein, and peripheral blood, or BM at the experiment end point, were analyzed for donor chimerism as described previously (Beaudin et al., 2014; Boyer et al., 2012; Boyer et al., 2011; Forsberg et al., 2006; Ooi et al., 2009; Smith-Berdan et al., 2011). Briefly, donor chimerism was assayed using inherent GFP or Tomato fluorescence, or CD45.1/CD45.2 alleles using antibodies to the CD45.1 (Ly5.1)-Alexa488 (BioLegend, Cat # 110718) and CD45.2 (Ly5.2)-Alexa700 (BioLegend, Cat # 109822) alleles and the lineage markers B220-APC-Cy7 (BioLegend, Cat # 103224), CD3-PE (BioLegend, Cat # 100308) or CD3-APC (eBioscience, Cat # 17-0031-82), Mac1-PECy7 (BioLegend, Cat # 101216), Ter119-PECy5 (BioLegend, Cat # 116210), Gr1-Pacific Blue (BioLegend, Cat # 108430) and CD61-Alexa 647 (BioLegend, Cat # 104314). B cells in PB were defined as $B220^{+}CD3^{-}Mac1^{-}Gr1^{-}Ter119^{-}$ cells; T cells as $CD3^{+}B220^{-}Mac1^{-}Gr1^{-}Ter119^{-}$ cells; GM cells as $Mac1^{+}Gr1^{+}B220^{-}CD3^{-}Ter119^{-}$ cells. When using UBC-GFP mice as donors, platelets and RBCs were isolated from peripheral blood without red cell hypotonic lysis. Donor platelets were defined as $GFP^{+}CD61^{+}Ter119^{-}FSc^{low}$, and donor RBCs were defined as $GFP^{+}Ter119^{+}FSc^{low}$. For CFU-S assays, lethally irradiated Wt or $Robo4^{-/-}$ mice were transplanted with 100 HSCs ($cKit^{+}/Lin^{-}/Sca1^{+}/Flk2^{-}/CD34^{-}$ BM cells) and numbers of spleen colonies were counted 12 days later, as described previously (Beaudin et al., 2014; Forsberg et al., 2006; Smith-Berdan et al., 2011).

BM homing assays. Wt BM cells were isolated from C57BL/6-Tg(UBC-GFP) mice (Schaefer et al., 2001) or from congenic wt mice and labeled with CFSE labeling dye (Invitrogen) for 5 minutes at room temperature, followed by antibody labeling and isolation of $cKit^{+}/Lin^{neg}/Sca1^{+}$/GFP+ or $cKit^{+}/Lin^{neg}/Sca1^{+}/CFSE^{hi}$ cells by two rounds of FACS. Recipient mice, lethally irradiated (1024 rads) 24 hours prior to transplantation, were injected IV with 40,000 - 60,000 cells in 100μl HBSS. Total blood was harvested by perfusion three hours post-transplant from individual mice, immediately followed by BM and spleen harvesting. Tissues were analyzed for the presence of CFSE-labeled or GFP+ cells by flow cytometry (Smith-Berdan et al., 2011). Graphs display the percent of total injected

cells recovered from each tissue, as described previously (Beaudin et al., 2014; Smith-Berdan et al., 2011).

Endothelial cell isolation and culturing. Endothelial cells were isolated from lungs, kidney or BM from wt or Robo4$^{-/-}$ mice and treated with collagenase I, as previously described (Dong et al., 1997; Jones et al., 2008; Smith-Berdan et al., 2012; Sobczak et al., 2010). Freshly isolated cells were used for gene and protein expression analyses. For CXCR4 analysis by flow cytometry, cells were stained with CXCR4-PE (eBioscience, Cat # 12-9991-81). For transwell assays, cells were enriched for endothelial cells by negative selection with anti-CD45 magnetic beads (Miltenyi) and by positive selection with anti-CD31 magnetic beads (Miltenyi). CD31-enriched cells were cultured for up to four passages under conditions promoting endothelial cell growth (Sobczak et al., 2010). Endothelial cells were stained with CD31-FITC, Sca1-PB, CD45.2-Alexa 700, Ter119-PECy5, VCAM1-PECy7 and Tie2-PE (Biolegend) for flow cytometric analysis (see above for details). For DiI-Ac-LDL analysis (Invitrogen), mice were injected IV with 10µg/mouse and endothelial cells were harvested 4 hours later and analyzed by flow cytometry for DiI-Ac-LDL staining. Two million events were collected for flow cytometry analysis, resulting in ~2,000 and 200 cells in the final gates for Figures 4C and 4E, respectively.

Transendothelial migration assays. Primary ECs were seeded onto 0.5% gelatin treated transwell inserts and grown to confluency. BM cells (lineage-depleted by magnetic selection, when appropriate) from wt or Robo4$^{-/-}$ mice were pre-incubated at 37º C for one hour, and then placed in the upper chamber of a transwell insert (5 um pore size). Bottom wells contained SDF1 (100 ng/ml) or IL-6 (100 ng/ml) (Weissenbach et al., 2004) as indicated. Cells were allowed to migrate for 2 hrs at 37º C before harvesting and analysis by flow cytometry, as described previously (Smith-Berdan et al., 2011). In some cases, permeability was induced by exposing starved cells to 2.4nM rhVEGF-165 for 3.5 hours or to 32µM histamine for 45 minutes prior to migration. For blocking assays, ECs were pretreated with anti-Itgα4 (clone PS/2; generously provided by Santa Cruz Biotechnologies) (Bowden

et al., 2002) or anti-CD31 (Pierce, Cat # ENMA3105, clone 2h8) (Bogen et al., 1994) antibodies at 10μg/ml for 30 minutes prior to cell migration towards SDF1. The antibodies utilized for HSC analysis were: cKit-PECy7 (Biolegend, Cat # 105814), Sca1-PB (Biolegend, Cat # 122520), Flk2-APC (BioLegend, Cat # 135310), CD34-FITC (eBioscience, Cat # 11-0341-85), PE-conjugated Lineage: CD3 (BioLegend, Cat # 100308), CD4 (BioLegend, Cat # 100408), CD5 (BioLegend, Cat # 100608), CD8 (BioLegend, Cat # 100708), B220 (BioLegend, Cat # 103208), Ter119 (BioLegend, Cat # 116208), Mac-1 (BioLegend, Cat # 101208), and Gr-1 (BioLegend, Cat # 108408).

In vitro permeability assays. Transwells with EC monolayers were prepared as above. FITC-Dextran (~40 kDa) or Cascade Blue-dextran (~10 kDa) was added to the top well at a final concentration of 50ug/ml. Fifteen minutes later, media was removed from the lower well and the amount of fluorescence was measured at 492/520 absorption/emission on a Victor X3 multilabel plate reader (Perkin Elmer). For histamine-induced passive permeability assays, cells were starved overnight; FITC-dextran was allowed to equilibrate through the EC layers for 5 minutes prior to stimulation with 32μM histamine for 15 minutes.

Vascular permeability assays. A modified Miles Assay was utilized to assess *in vivo* vascular permeability (Miles and Miles, 1952). Mice were injected IV with Evans Blue (50mg/kg), and then euthanized by isoflurane inhalation. Vascular leak was determined by isolating tissues 5-10 minutes post injection and measuring Evans Blue absorbance, expressed as OD650/tissue mass. For induced permeability assays, VEGF (2μg/mouse) was injected once IV followed by Evans Blue dye 5 minutes later. The dye was allowed to leak into the tissues for an additional 15 minutes prior to tissue harvest, whereas histamine (100μg/mouse) was injected IV three times 5 minutes apart, followed by Evans Blue dye IV 5 minutes later, and 5 minutes prior to tissue harvest. For radiation permeability assays, mice were treated with a lethal dose of radiation 3 days prior to Miles assay.

Immunohistochemistry. Bones were embedded into OCT cryopreservation media on an ethanol/dry ice slurry immediately after dissection from Wt or Robo4$^{-/-}$ mice, and stored at -80°C. 7 micron BM sections were cut using a tungsten blade, fixed with acetone at -20°C for 10 minutes or with 4% paraformaldehyde at 4°C for 20 minutes. Sections were blocked with 10% new goat serum prior to overnight antibody staining at 4°C followed by incubation for 1 hour with fluorescently conjugated secondary antibodies. The primary antibodies used were α-laminin-PGP (Sigma, Cat # L9393), α-VCAM1-PGP (BioLegend, Cat # 105710). The secondary antibodies included Goat α-rabbit –Alexa 594 (Invitrogen, Cat # A11037), Goat α-rat – Dylight 488 (BioLegend, Cat # 405409). Samples were imaged with a Keyance Microscope and images were quantified with ImageJ software by a sample-blind technician.

Mobilization. Mice were injected with either histamine (5mg/kg; IV), or with AMD3100 (5mg/kg; subcutaneously) and/or rhVEGF-165 (2μg/mouse; IV), as indicated. Total blood was isolated by perfusion with PBS/20 mM EDTA and processed for cell counts and flow cytometry analysis to determine the numbers and frequencies of each cell population, as described previously (Smith-Berdan et al., 2011). Reconstitution assays from mobilized blood were performed by transplanting ½ of the blood mouse equivalent into a lethally irradiated host (1024 rads). Recipient mice were bled at the indicated intervals posttransplantation via the tail vein, and peripheral blood was analyzed for donor chimerism as described above and previously (Boyer et al., 2012; Boyer et al., 2011; Forsberg et al., 2006; Ooi et al., 2009; Smith-Berdan et al., 2011). The antibodies utilized for HSC, MPP, Myeloid Progenitors, and mature cell analysis were: cKit-PECy7 (Biolegend, Cat # 105814), Sca1-PB (Biolegend, Cat # 122520), Flk2-PE (eBioscience, Cat # 12-1351-83), Alexa-488 or FITC conjugated Lineage: CD3 (BioLegend, Cat # 100306), CD4 (BioLegend, Cat # 100423), CD5 (BioLegend, Cat # 100612), CD8 (BioLegend, Cat # 100723), B220 (BioLegend, Cat # 103225), Ter119 (BioLegend, Cat # 116215), Mac-1 (BioLegend, Cat # 101217), and Gr-1 (BioLegend, Cat # 108417), and CD27-APC (BioLegend, Cat # 124212).

Gene expression. Quantitative RT-PCR was performed on freshly isolated BM endothelial cells as described previously (Forsberg et al., 2005; Forsberg et al., 2006; Smith-Berdan et al., 2011; Smith-Berdan et al., 2012) using a ViiA7 Real Time PCR System (Life Technologies) and Quantace SensiMixPlus SYBR (BioLine). Expression of β-actin was used to normalize cDNA amounts between samples. mVEGFR2 and mVEGFR3 primers were from Primer Depot.

mCXCR4 forward : AGCCTGTGGATGGTGGTGTTTC, reverse: CCTTGCTTGATGACTCCAAAAG

mβ-actin forward: GACGGCCAGGTCATCACTAT, reverse: CGGATGTCAACGTCACACTT

mVEGFR2 forward: TCCAGAATCCTCTTCCATGC, reverse: CCAGAGACCCTCGTTTTCAG

mVEGFR3 forward: CCCTGCAGGATATGGATAGG, reverse: GCTCTGCCTCGGACTCCT

mRobo4 forward: CAGCCTGGTTAGCTCTTCTGATG, reverse: GCACGAGCAAAGTGAGTATCAGC

mVCAM1 forward: TTGGGAGCCTCAACGGTACT, reverse: GCAATCGTTTTGTATTCAGGGGA

mVE-cadherin forward: TCTTGCCAGCAAACTCTCCT, reverse: TTGGAATCAAATGCACATCG

SUPPLEMENTAL REFERENCES

Beaudin, A.E., Boyer, S.W., and Forsberg, E.C. (2014). Flk2/Flt3 promotes both myeloid and lymphoid development by expanding non-self-renewing multipotent hematopoietic progenitor cells. Exp Hematol *42*, 218-229 e214.

Bogen, S., Pak, J., Garifallou, M., Deng, X., and Muller, W.A. (1994). Monoclonal antibody to murine PECAM-1 (CD31) blocks acute inflammation in vivo. J Exp Med *179*, 1059-1064.

Bowden, R.A., Ding, Z.M., Donnachie, E.M., Petersen, T.K., Michael, L.H., Ballantyne, C.M., and Burns, A.R. (2002). Role of alpha4 integrin and VCAM-1 in CD18-independent neutrophil migration across mouse cardiac endothelium. Circ Res *90*, 562-569.

Boyer, S.W., Beaudin, A.E., and Forsberg, E.C. (2012). Mapping differentiation pathways from hematopoietic stem cells using Flk2/Flt3 lineage tracing. Cell Cycle *11*, 3180-3188.

Boyer, S.W., Schroeder, A.V., Smith-Berdan, S., and Forsberg, E.C. (2011). All hematopoietic cells develop from hematopoietic stem cells through Flk2/Flt3-positive progenitor cells. Cell Stem Cell *9*, 64-73.

Broxmeyer, H.E., Orschell, C.M., Clapp, D.W., Hangoc, G., Cooper, S., Plett, P.A., Liles, W.C., Li, X., Graham-Evans, B., Campbell, T.B., *et al.* (2005). Rapid mobilization of murine and human hematopoietic stem and progenitor cells with AMD3100, a CXCR4 antagonist. J Exp Med *201*, 1307-1318.

Dong, Q.G., Bernasconi, S., Lostaglio, S., De Calmanovici, R.W., Martin-Padura, I., Breviario, F., Garlanda, C., Ramponi, S., Mantovani, A., and Vecchi, A. (1997). A general strategy for isolation of endothelial cells from murine tissues. Characterization of two endothelial cell lines from the murine lung and subcutaneous sponge implants. Arteriosclerosis, thrombosis, and vascular biology *17*, 1599-1604.

Forsberg, E.C., Prohaska, S.S., Katzman, S., Heffner, G.C., Stuart, J.M., and Weissman, I.L. (2005). Differential expression of novel potential regulators in hematopoietic stem cells. PLoS Genet *1*, e28.

Forsberg, E.C., Serwold, T., Kogan, S., Weissman, I.L., and Passegue, E. (2006). New evidence supporting megakaryocyte-erythrocyte potential of flk2/flt3+ multipotent hematopoietic progenitors. Cell *126*, 415-426.

Jones, C.A., London, N.R., Chen, H., Park, K.W., Sauvaget, D., Stockton, R.A., Wythe, J.D., Suh, W., Larrieu-Lahargue, F., Mukouyama, Y.S., *et al.* (2008). Robo4 stabilizes the vascular network by inhibiting pathologic angiogenesis and endothelial hyperpermeability. Nat Med *14*, 448-453.

Liles, W.C., Broxmeyer, H.E., Rodger, E., Wood, B., Hubel, K., Cooper, S., Hangoc, G., Bridger, G.J., Henson, G.W., Calandra, G., *et al.* (2003). Mobilization of hematopoietic progenitor cells in healthy volunteers by AMD3100, a CXCR4 antagonist. Blood *102*, 2728-2730.

Miles, A.A., and Miles, E.M. (1952). Vascular reactions to histamine, histamine-liberator and leukotaxine in the skin of guinea-pigs. J Physiol *118*, 228-257.

Muzumdar, M.D., Tasic, B., Miyamichi, K., Li, L., and Luo, L. (2007). A global double-fluorescent Cre reporter mouse. Genesis *45*, 593-605.

Ooi, A.G., Karsunky, H., Majeti, R., Butz, S., Vestweber, D., Ishida, T., Quertermous, T., Weissman, I.L., and Forsberg, E.C. (2009). The adhesion molecule esam1 is a novel hematopoietic stem cell marker. Stem Cells *27*, 653-661.

Schaefer, B.C., Schaefer, M.L., Kappler, J.W., Marrack, P., and Kedl, R.M. (2001). Observation of antigen-dependent CD8+ T-cell/ dendritic cell interactions in vivo. Cellular immunology *214*, 110-122.

Smith-Berdan, S., Nguyen, A., Hassanein, D., Zimmer, M., Ugarte, F., Ciriza, J., Li, D., Garcia-Ojeda, M.E., Hinck, L., and Forsberg, E.C. (2011). Robo4 cooperates with CXCR4 to specify hematopoietic stem cell localization to bone marrow niches. Cell Stem Cell *8*, 72-83.

Smith-Berdan, S., Schepers, K., Ly, A., Passegue, E., and Forsberg, E.C. (2012). Dynamic expression of the Robo ligand Slit2 in bone marrow cell populations. Cell Cycle *11*, 675-682.

Sobczak, M., Dargatz, J., and Chrzanowska-Wodnicka, M. (2010). Isolation and culture of pulmonary endothelial cells from neonatal mice. Journal of visualized experiments : JoVE.

Weissenbach, M., Clahsen, T., Weber, C., Spitzer, D., Wirth, D., Vestweber, D., Heinrich, P.C., and Schaper, F. (2004). Interleukin-6 is a direct mediator of T cell migration. European journal of immunology *34*, 2895-2906.
